# PI-Informer-Based Multi-Step Prediction of Side-Wall Root Weld Width for Ultra-Narrow Gap Arc Welding

**DOI:** 10.3390/s26216974

**Published:** 2026-11-04

**Authors:** Jing Ma, Aihua Zhang, Yongqiang Ma, Liang Zhu

**Affiliations:** 1 School of Automation and Electrical Engineering, Lanzhou University of Technology, Lanzhou 730050, China; 2 School of Microelectronics Industry Education Integration, Lanzhou University of Technology, Lanzhou 730050, China; 3 School of Materials Science and Engineering, Lanzhou University of Technology, Lanzhou 730050, China

**Keywords:** ultra-narrow gap welding, side-wall root weld width, multi-step prediction, physics-informed learning, informer, intelligent monitoring

## Abstract

**Highlights:**

This work develops a physics-informed Improved Informer (PI-Informer) for multi-step prediction of side-wall root weld width in ultra-narrow gap arc welding. A physical residual loss function and multi-scale 1D-CNN collaborative architecture are proposed, and systematic ablation and comparative experiments are conducted to verify model performance under multiple prediction horizons.

**What are the main findings?**

- The introduced physical-residual loss achieves quantitative embedding of welding thermal-balance prior knowledge; the optimal weight *β* = 0.05 yields the best trade-off between prediction accuracy and physical consistency.
- The collaborative architecture combining multi-scale 1D-CNN and improved decoder start-tokens achieves multi-source fusion among local transient features of welding electrical signals, global temporal dependencies of molten-pool heat accumulation, and recent welding states, thereby effectively reducing multi-step prediction errors.

**What are the implications of the main findings?**

- The multi-step prediction paradigm covering three typical prediction horizons of *t*, *t* + 3, *t* + 15 supports look-ahead trend prediction of weld geometry, satisfying the look-ahead regulation demands of intelligent welding systems.
- The proposed framework provides a feasible technical reference for intelligent perception and closed-loop quality control of ultra-narrow gap arc welding.

**Abstract:**

Multi-step prediction of side-wall root weld width is essential for intelligent monitoring and closed-loop quality control in ultra-narrow gap arc welding. To address limited physical interpretability of pure data-driven models and insufficient control response time from single-step prediction, a Physics-Informed Informer (PI-Informer) multi-step prediction model constrained by welding thermal-balance mechanisms is proposed in this work. Thermal prior knowledge is embedded into training via a physically constrained loss function to compensate for physical deficiencies in pure data-driven learning. Local transient features are extracted from welding electrical signals by a multi-scale 1D-CNN and further integrated with global temporal dependencies of molten-pool heat accumulation through the Informer encoder. Unlike the standard Informer, decoder initialization is implemented with fused features of electrical signals and process parameters, enabling multi-step prediction at three typical prediction horizons of *t*, *t* + 3, and *t* + 15. From ablation experiments, the optimal weight *β* = 0.05 is identified for the best trade-off between accuracy and physical consistency, and multi-step errors are effectively mitigated by the 1D-CNN module. Under identical conditions, PI-Informer attains lower RMSE, MAE and MAPE than vanilla Informer, GRU and LSTM across evaluation metrics, and yields the lowest batch-averaged physical residual among all compared models. A favourable balance between accuracy and physical interpretability is achieved, and a feasible solution is provided for intelligent perception and proactive quality control in ultra-narrow gap welding.

## 1. Introduction

Ultra-narrow gap welding has become a promising advanced welding technology for high-end heavy-plate equipment manufacturing owing to its low heat input, high efficiency and excellent mechanical properties of welded joints [1,2]. Achieving reliable side-wall root fusion is regarded as a critical challenge for forming-quality control in ultra-narrow gap arc welding [3,4]. The side-wall root weld width is adopted as a key geometric metric for evaluating root fusion status. Due to the confined spatial environment inside the weld groove, direct non-destructive online measurement of this metric remains difficult to implement in practical applications [5,6]. Process-driven predictive modelling offers a viable alternative to address the bottleneck of online monitoring for such weld-formation parameters [7,8]. Most existing studies perform single-step prediction of weld geometry using historical welding data. Nevertheless, weld formation is a strongly nonlinear time-varying process governed by coupled molten-pool heat accumulation and heat dissipation, which exhibits notable dynamic hysteresis. Combined with inherent response lags within welding control hardware, closed-loop quality regulation requires advance knowledge of short-term geometric trends to reserve sufficient time for adjusting process parameters. Accordingly, developing multi-step prediction approaches for side-wall root weld width is practically meaningful for advancing intelligent ultra-narrow gap welding systems and forms a necessary basis for online monitoring and active quality regulation.

Prediction of weld geometric features is an active research topic within intelligent welding-quality monitoring [9,10], where data-driven modelling has become the dominant technical pathway. Many studies construct weld-prediction models by feeding multi-source process measurements, such as welding electrical signals, arc acoustic signals and molten-pool images, into machine-learning frameworks [11,12,13,14,15]. Progress in deep learning has further promoted the adoption of CNN- and LSTM-based networks for welding time-series feature extraction and weld-geometry forecasting [16,17]. The Informer architecture, which is well-suited for capturing long-range temporal dependencies, has achieved competitive performance across time-series tasks including power-system and traffic forecasting [18,19,20]. Fan et al. adopted the Informer for laser-MIG hybrid welding, achieving single-step prediction of top-side and back-side weld widths with image-derived temporal features [21]. Even so, such works are limited to single-step outputs and cannot satisfy the multi-step look-ahead requirements of closed-loop welding control. Furthermore, these purely data-driven models incorporate no physical welding constraints. Although they possess strong nonlinear fitting capacity, their performance is heavily dataset-dependent. Under operational disturbances, physically implausible outliers and large prediction deviations may occur [22], leading to limited generalization and poor interpretability for industrial deployment.

Integrating physical prior knowledge with data-driven models provides a feasible way to mitigate the above drawbacks, and preliminary attempts have been reported in welding-related modelling [23,24,25,26,27]. Embedded physical principles such as fluid dynamics, pressure balance and heat conduction can improve model accuracy and stability, representing a growing research frontier for intelligent welding monitoring. However, existing physics-enhanced welding models are mostly designed for single-step instantaneous prediction. Short-term multi-step prediction targeting side-wall root forming under ultra-narrow gap conditions remains under-explored.

For side-wall root weld width prediction in ultra-narrow gap arc welding, pure data-driven models lack physical welding constraints and exhibit limited interpretability. Meanwhile, conventional single-step prediction is incapable of satisfying the look-ahead regulation demands of intelligent welding systems. Combined with welding thermal-balance and heat-transfer laws, this study integrates the advantages of multi-scale temporal feature extraction and long-sequence modelling, and a Physics-Informed Informer (PI-Informer) multi-step prediction model with physical constraints is proposed accordingly. The framework consists of a data-driven branch and a physical-constraint branch. Within the data-driven branch built upon the Informer backbone architecture, multi-scale 1D-CNN is introduced to extract local transient features from welding electrical signals. ProbSparse self-attention is adopted to capture long-range global temporal dependencies of molten-pool heat accumulation, and the improved decoder start tokens are utilized to perceive recent welding states for multi-step output of side-wall root weld width. For the physical-constraint branch, outputs derived from the thermal-balance mechanism are incorporated into model training as regularization loss terms, so that the network is constrained jointly by measured samples and welding physical priors. Multi-step prediction of side-wall root weld width is carried out for three typical forecast horizons including *t*, *t* + 3, and *t* + 15. A series of ablation and comparative experiments are performed to verify model performance and the function of each module.

The remainder is organized as follows: Section 2 presents the experimental platform and data-acquisition scheme. Section 3 establishes the heat-balance mechanism model for side-wall root weld width. Section 4 details the proposed PI-Informer model, including network architecture, physical-constraint design, training procedure and online prediction workflow. Section 5 describes comparative and ablation experiments as well as model validation results. Section 6 concludes this paper and discusses future work.

## 2. Experiment Setup

### 2.1. Ultra-Narrow Gap Arc Welding Experimental System

**Figure 1:**
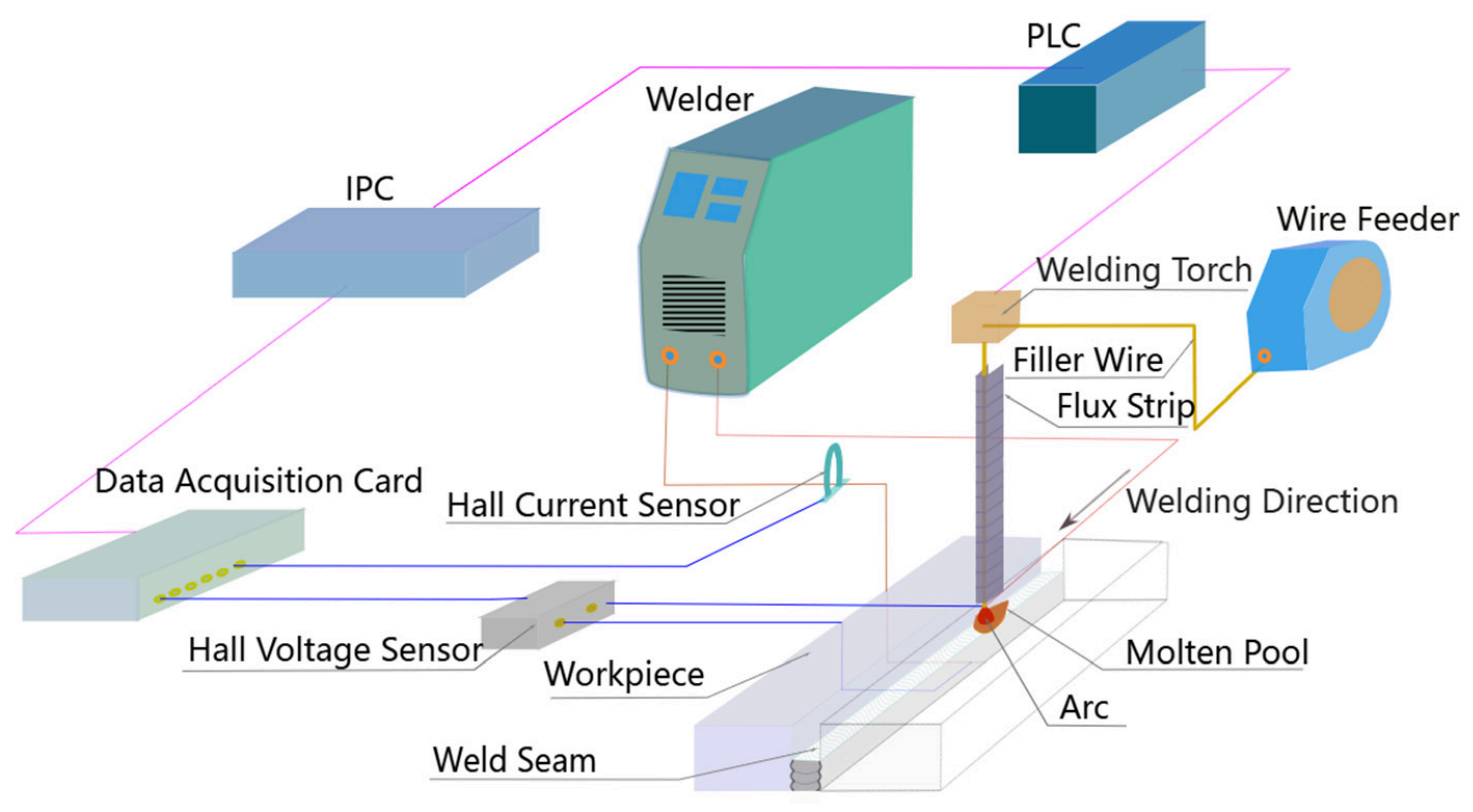
Experimental equipment for the UNG-AW.

Ultra-narrow gap arc welding (UNG-AW) is an efficient and low-heat-input single-pass forming welding technique that exhibits prominent advantages in the manufacturing of heavy-plate high-end equipment [27]. The UNG-AW experimental platform adopted in this study consists of an inverter arc welding power supply, a wire-strip cooperative feeding mechanism, a dedicated welding torch, a PLC welding control system (FX3U-64MR and FX3U-4DA, Mitsubishi Electric Corporation, Tokyo, Japan), a servo-driven motion platform, an electrical signal acquisition system, and an industrial control computer, as illustrated in Figure 1. The real-time welding electrical signals are captured by a Hall current sensor (LHB-600A5VD2Y4, Zhuhai Xinghui Electronics Technology Co., Ltd., Zhuhai, China) and a Hall voltage sensor (KDV-100V, Zhuhai Xinghui Electronics Technology Co., Ltd., Zhuhai, China), with a high-speed NI USB-6251 (National Instruments Corporation, Austin, TX, USA) data-acquisition card responsible for signal recording. In the welding experiments, H08Mn2Si (Tianjin Golden Bridge Welding Materials Group Co., Ltd., Tianjin, China) low-alloy steel wire with a radius of 0.6 mm was used as the filling material, and Q235B (Baoshan Iron & Steel Co., Ltd., Shanghai, China) low-carbon steel plates with a thickness of 30 mm were adopted as the base material. The welding process was implemented at constant welding speed with synchronized wire and strip feeding. The feeding ratio of wire to strip was set to 1:55, and the distance between the wire tip and the groove bottom was maintained at 1.5 mm. During the UNG-AW process, the electric energy is converted into thermal energy by the welding arc to melt the base metal, filler wire, and flux strip. The molten metal transfers to the molten pool in the form of droplets under electromagnetic contraction force and finally forms a solid weld bead after cooling and solidification.

**Figure 2:**
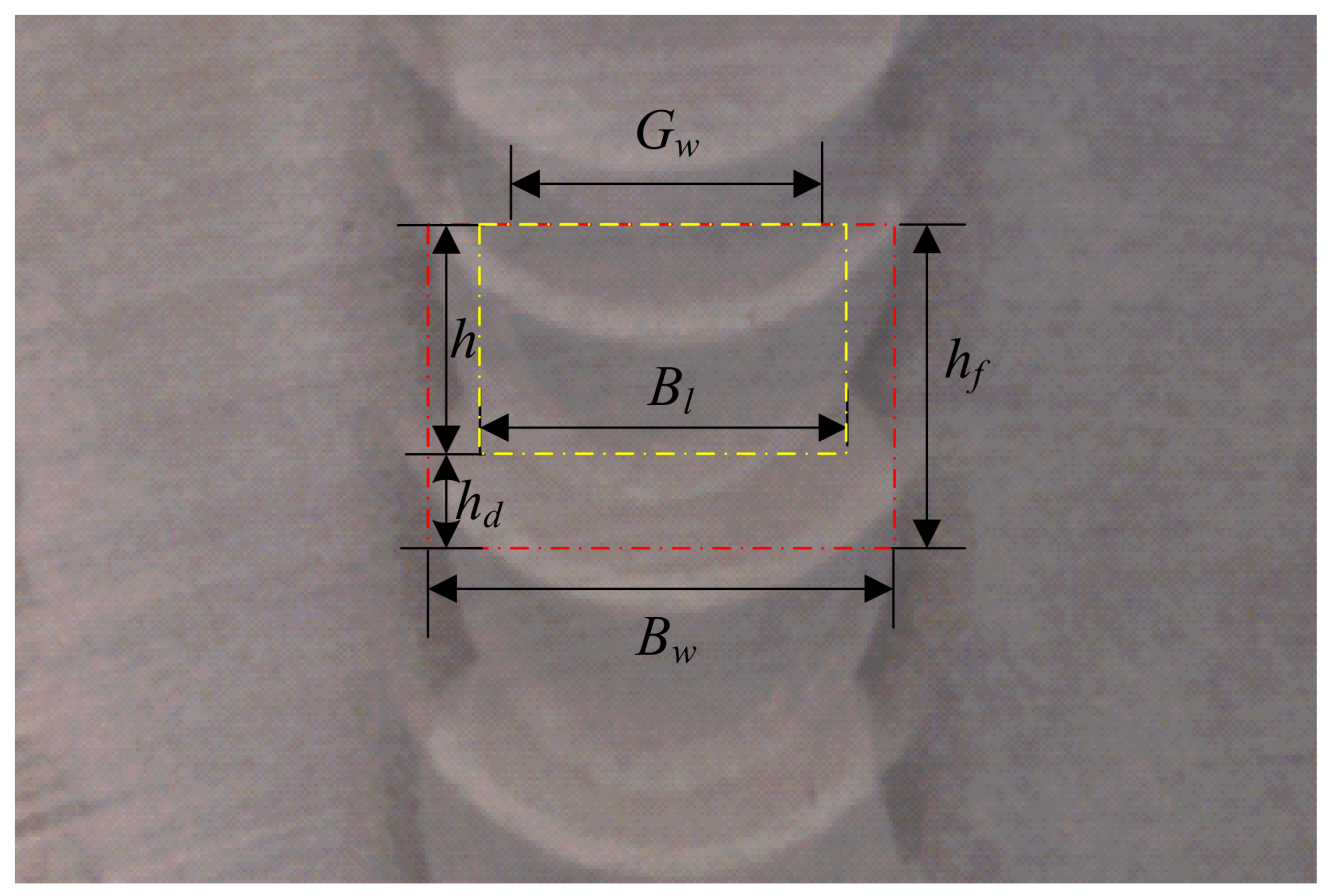
Geometric parameters of the weld fusion zone.

**Figure 3:**
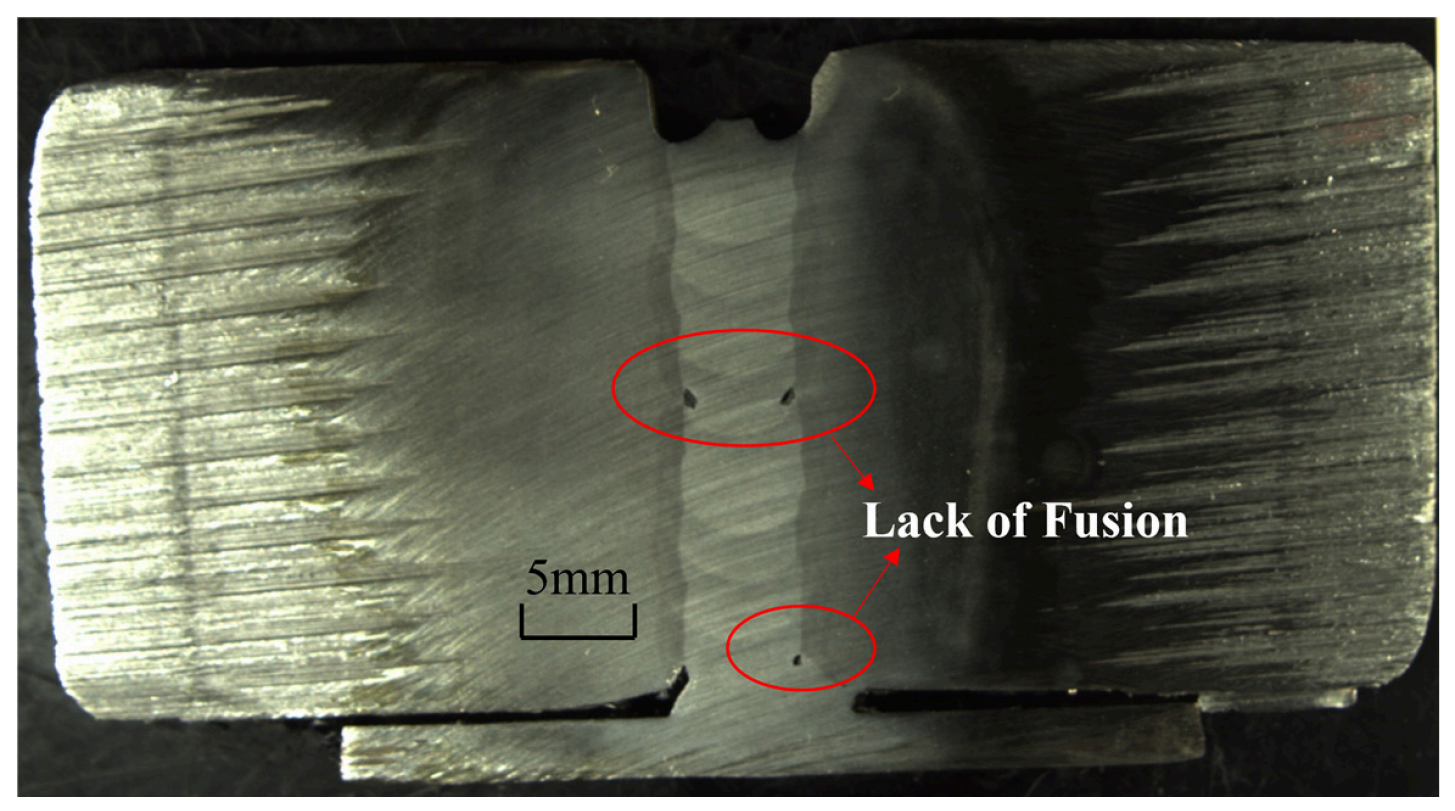
Typical cross-sectional morphology of a weld with side-wall root lack-of-fusion.

The geometric parameters of the weld fusion zone are defined in Figure 2, where *G_w_* denotes the groove gap width, *B_w_* is the maximum weld width, *B_l_* represents the side-wall root weld width, *h_f_* is the weld penetration depth, and *h* is the filling height of the weld layer. Effective side-wall root fusion is achieved only when *B_l_* > *G_w_*, indicating that *B_l_* is the core indicator for evaluating root fusion quality. To quantitatively characterize the weld forming state, two dimensionless evaluation indices are defined in this work: the side-wall width ratio *κ* = *B_l_*/*B_w_* and the layer height ratio χ = *h*/*h*_f_. *κ* quantifies the U-shaped feature of the weld cross-section, while *χ* characterizes the inter-pass fusion state. A sound U-shaped weld with full side-wall root fusion satisfies *κ* > *G_w_*/*B_w_*. Otherwise, a V-shaped weld with a high risk of incomplete fusion tends to form. Under fixed process parameters, *κ* is governed by the side-wall root weld width *B_l_*, and a larger *κ* corresponds to better side-wall root fusion. The layer height ratio is defined as *χ* = *h*/*h_f_*, where the penetration depth *h_f_* consists of the filling height of the weld layer *h* and the remelting depth *h_d_* of the current pass into the previous weld pass. Satisfactory inter-pass fusion requires *h_d_* >0, which yields *h_f_* >*h* and *χ* < 1. Therefore, a desirable U-shaped weld with full side-wall fusion is achieved when *κ* > *G_w_*/*B_w_* and *χ* < 1; otherwise, a V-shaped weld featuring side-wall root lack-of-fusion tends to occur. Typical cross-sectional morphology of welds with side-wall root lack-of-fusion is shown in Figure 3. Accordingly, the forming quality of the side-wall root is strongly dependent on *B_l_*, *κ* and χ.

Typical cross-sectional morphology of welds featuring side-wall root lack-of-fusion is shown in Figure 3. Therefore, the side-wall root forming quality is closely dependent on both *κ* and χ.

### 2.2. Data Acquisition and Sample Construction

During the UNG-AW welding process, real-time welding current and arc voltage signals were synchronously collected by Hall sensors at a sampling frequency of 50 kHz. After welding, the welded plate was sliced perpendicular to the weld direction with an interval of 4.0–5.0 mm. Each slice incurred a machining loss of approximately 1.2 mm. After polishing and corrosion treatment, the cross-sectional geometric parameters of each slice were precisely measured and rearranged according to the actual welding direction. Consequently, the spatially and temporally matched weld forming parameters, including side-wall root weld width *B_l_*, side-wall width ratio *κ*, and layer height ratio χ, were obtained corresponding to the original electrical signals.

In total, 12 stable welded specimens were manufactured in this study. Each specimen contains 8–10 independent physical weld-bead passes. Each valid weld pass possesses 150 mm usable weld length. After metallographic slicing, 24–28 physical cross-section slices can be harvested per weld pass. Two side-wall-root-fusion-width measurements (left-side and right-side groove) are obtained from every single physical slice. After filtering slices containing pores, slag inclusions or severe forming defects, 1200 high-quality valid sliding-window time-series samples are retained for modelling.

Each averaging operation over 2500 raw 50 kHz sampling points yields one effective time-series step with physical duration of 50 ms. Accordingly, *t* represents the current moment, *t* + 3 corresponds to 150 ms ahead, *t* + 15 corresponds to 750 ms ahead.

After welding, the workpiece is sectioned perpendicularly to the weld seam with a step size of 5 mm. Since the arc is unstable at the arc-start and arc-end regions, this work focuses on modelling and predicting the side-wall root fusion state for the stable welding segment without arc climbing. Therefore, the slices from the start and end of the weld are discarded. Let the total weld length be *L*, and the discarded lengths at the two ends be *L*_A_ and *L*_B_, respectively. The effective weld length is defined as *L*_e_ = *L* − *L*_A_ − *L*_B_. The cutting step size *d*_s_ denotes the distance between the same-side surfaces of adjacent slices, which already accounts for kerf loss from sawing. The number of effective slices is calculated as *M*_e_ *= L*_e_/*d*_s_ + 1. In our experiments, the total weld length L of each specimen ranges from approximately 140 to 150 mm. Around 5 mm is discarded from each end of the weld, and the cutting step size is set to *d*_s_ = 5 mm. Accordingly, 24 to 28 valid slices can be obtained for a single weld pass. Cross-sectional parameters on the left and right sides are measured for each slice and sorted chronologically by welding time, yielding a total of 48 to 56 valid measurements per weld pass.

In addition, there exists system latency Δ*t* = *t*_0_ − *t*_s_ from the start of effective electrical-signal acquisition *t*_s_ to the actual movement start moment *t*_0_ of the welding torch. This latency includes arc-ignition delay, PLC scanning and relay action time, and servo enable time. We adopt *t*_0_ (the real starting instant of torch movement) as the unified time reference, and establish the time–space mapping between welding signals and metallographic slices.

Ultra-narrow gap arc welding is carried out at a constant speed. The displacement of the welding gun along the welding direction and the working time meet the following requirements:

*L* = *V* ⋅ (*t* − *t*_0_) (1)

where *V* is welding speed, *L* is torch displacement along the welding direction. The spatial position of the k-th metallographic slice is expressed as

*L_k_* = *L*_0_ + *k* ⋅ *d_s_* (2)

where *L*_0_ is the position of the first slice, *d*_s_ denotes the slice step size (distance between adjacent slices), *L*_k_ is the spatial position of the k-th metallographic cross-section slice. Given known welding speed and *t*_0_, the welding instant corresponding to each metallographic slice can be reversely derived from its spatial coordinate.

**Figure 4:**
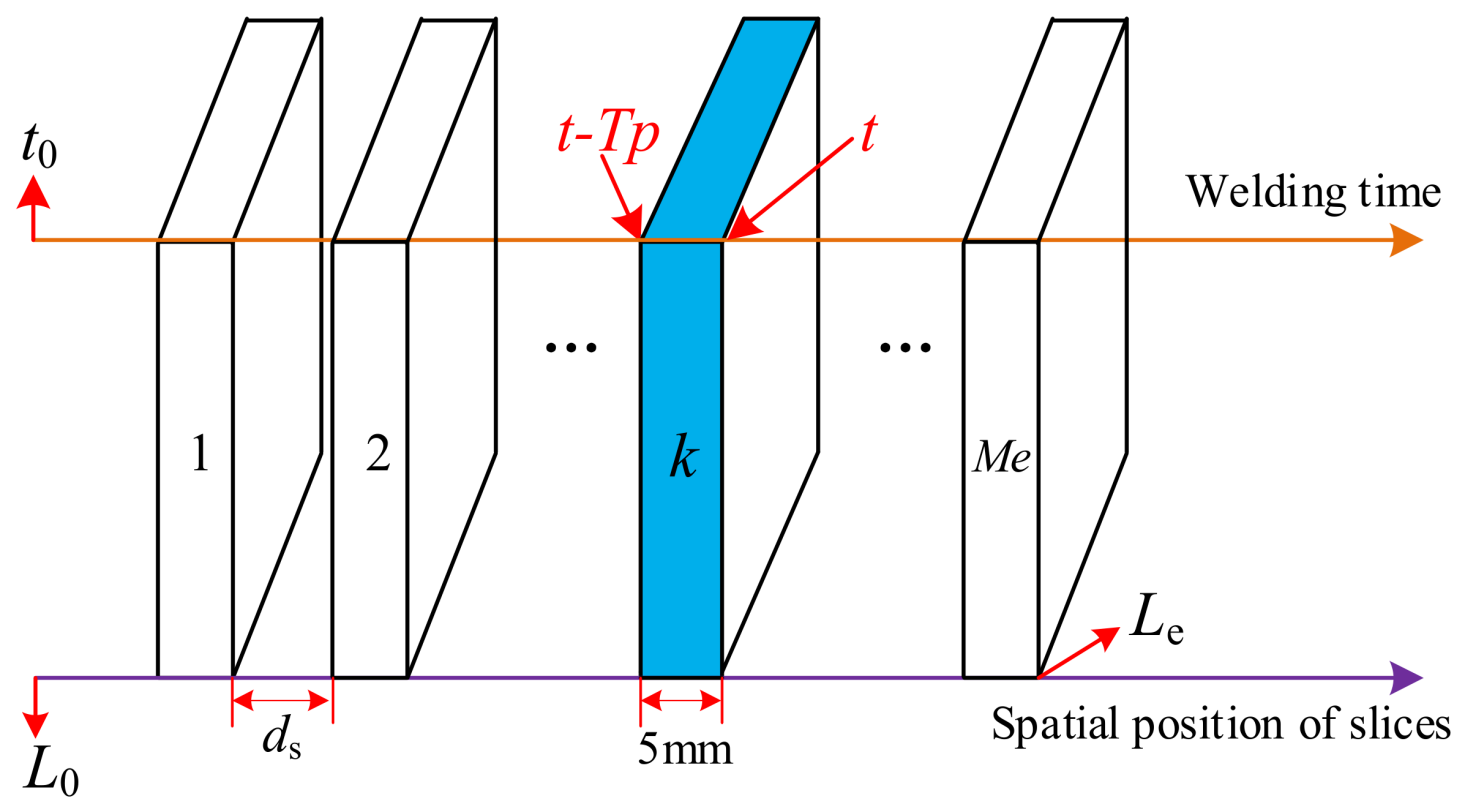
Time–space mapping of welding signals and slices.

This time–space correspondence workflow is further illustrated in Figure 4. This time–space alignment workflow unifies weld-slice geometric parameters and welding electrical signals under the same time–space framework, supporting data back-tracking for modelling. Since metallographic measurements are discrete spatial snapshots obtained by post-weld destructive slicing rather than continuous in-process time-series, no interpolation is performed for geometric labels. Each slice-derived geometric value is strictly assigned to its corresponding reconstructed time instant t. The multi-step ground-truth targets for *t* + 3 and *t* + 15 are extracted from subsequent downstream physical slices located further along the welding travel direction.

## 3. Mechanism Model Based on Welding Thermal-Balance Principle

The heat transferred from the welding arc to the weld joint follows thermal-balance constraints among arc heat input, heat absorbed by molten metal and various heat-loss terms. In this section, an analytical mechanism model for side-wall root weld width is derived based on thermal-balance theory.

The effective linear heat input *Q*(*t*) imposed by the arc at time *t* is formulated as

(3) $$Q(t)=\frac{\mathrm{\eta U}(t)I(t)}{V(t)}$$

According to thermal-balance law, the effective heat input from the arc equals the heat absorbed by weld metal [26], which yields

(4) $$\frac{\eta_{1}U(t)I(t)}{V(t)}=S(t)h_{m}\rho$$

where *η*_1_ denotes the thermal-efficiency coefficient; *U*(*t*), *I*(*t*) and *V*(*t*) represent arc voltage, welding current and welding speed at time instant t, respectively. *h_m_* is the specific enthalpy of weld metal, *ρ* is the material density, and *S*(*t*) stands for the area of weld fusion zone at time *t*.

**Figure 5:**
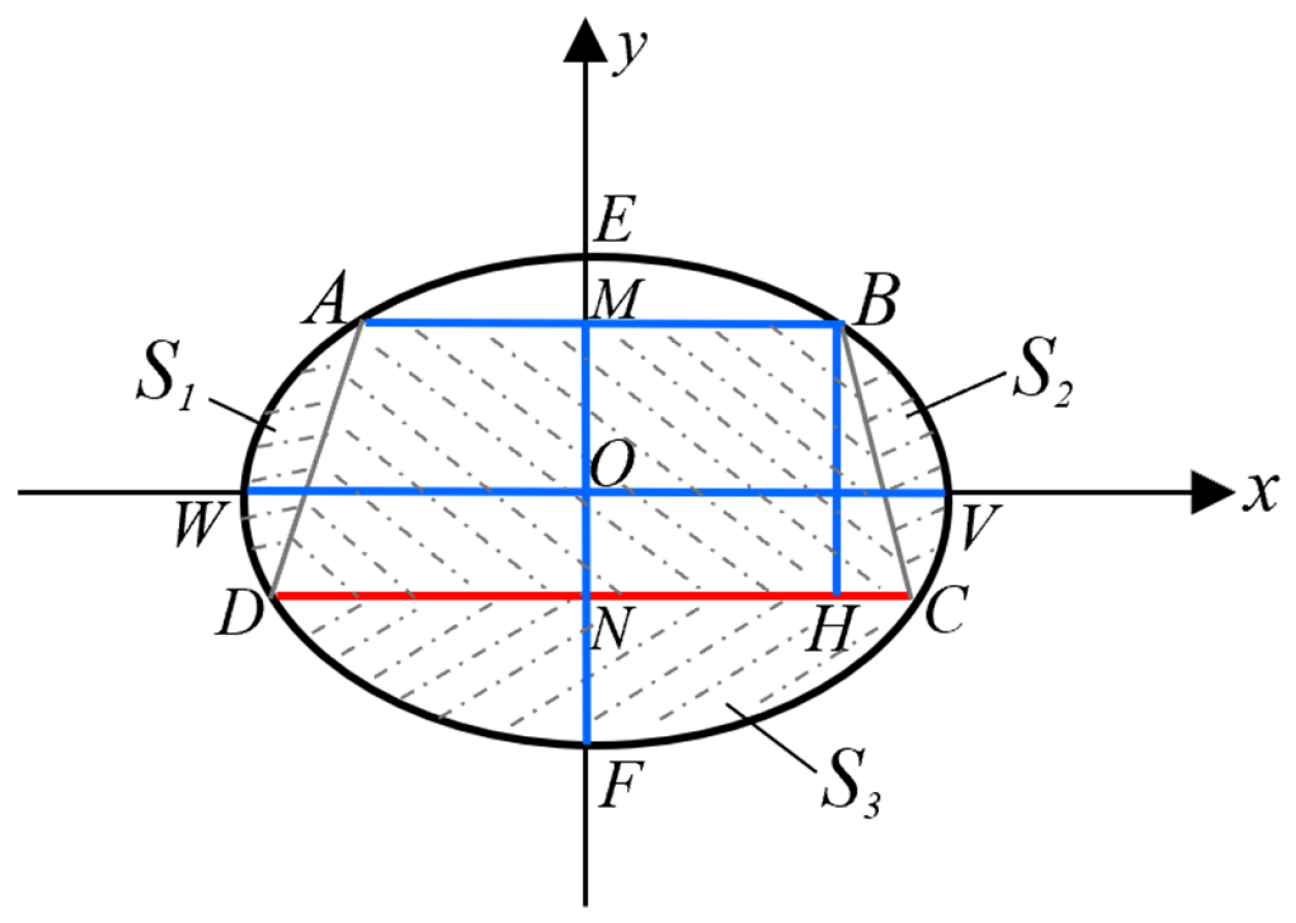
Weld seam cross-sectional molten-pool contour area model.

Ultra-narrow gap arc welding produces three types of weld profiles: flat, concave, and convex, among which the flat profile is considered the ideal form. Based on the actual cross-sectional morphology of ultra-narrow gap arc welding, this paper establishes a weld pool area model for flat-weld cross-sections. Previous researchers have conducted mathematical modelling and analysis of weld-bead profiles in single-pass, multi-layer arc welding and demonstrated that the semi-elliptical function model achieves the highest fitting accuracy for the topmost bead contour [26]. In this work, the molten-pool contour on the weld cross-section of ultra-narrow gap arc welding is approximated as a closed curve composed of the elliptical arc and the straight-line segment AB, as illustrated in Figure 5. This curve is expressed as

(5) $$\frac{x^{2}}{a^{2}}+\frac{y^{2}}{b^{2}}=1$$

In Figure 5, $\bar{\mathrm{AB}}=G_{W}$, $\bar{\mathrm{CD}}=B_{l}$, $\bar{\mathrm{WV}}=B_{W}$, $\bar{\mathrm{MN}}=h$, and $\bar{\mathrm{MF}}=h_{f}$, $\bar{\mathrm{CD}}-\bar{\mathrm{AB}}=\delta$. The fusion-zone area *S*(*t*) on the weld transverse cross-section is approximately calculated by

*S* = *S*_1_ + *S*_2_ + *S*_3_ + *S*_4_ (6)

Here, *S*_1_ and *S*_2_ denote the bilateral elliptical-segment areas, *S*_3_ represents the bottom elliptical-segment area, and *S*_4_ is the central trapezoidal weld zone area. Given the sub-area integral formulas and flat-weld approximation conditions $a\approx \frac{B_{w}}{2},b\approx \frac{h_{f}}{2}$, the area of the weld fusion zone *S*(*t*) can be approximately considered as

(7) $$S\approx \frac{B_{l}h}{4\kappa \chi }\left\{4\chi \left(\chi -1\right)\sqrt{\left(\frac{1}{\chi }-1\right)}+\left(1.6+\kappa \sqrt{1-\kappa^{2}}\right)+5\frac{G_{w}}{h}\left[\mathrm{arcsin\kappa}-\arcsin(1-2\chi )\right]+\left(2\kappa +2\chi -4\kappa \chi \right)\right\}-\frac{1}{2}G_{w}h$$

Under steady-state welding conditions, the geometry of fusion zone can be approximated as a semi-ellipse with the maximum weld width as the major axis and penetration depth as the minor axis. The previously defined dimensionless coefficients, side-wall width ratio *κ* and layer height ratio χ, are adopted to calculate fusion-zone area. Substituting the area expression into Equation (4), the analytical mechanism model for side-wall root weld width can be derived:

(8) $$\begin{array}{l} B_{l\_\mathrm{mec}}(t)\\ =\frac{8\kappa \chi \eta_{3}G_{w}\left[\frac{2\eta_{1}U(t)I(t)}{\pi \eta_{2}r^{2}\rho h_{m}V_{f}(t)}+1\right]}{\left\{4\chi \left(\chi -1\right)\sqrt{\left(\frac{1}{\chi }-1\right)}+1.6+\kappa \sqrt{1+\kappa^{2}}+2\left(\kappa +\chi \right)-4\kappa \chi +5\frac{G_{w}^{2}V(t)}{\eta_{2}\pi r^{2}V_{f}(t)}\left[\arcsin\kappa -\arcsin(1-2\chi )\right]\right\}} \end{array}$$

Equation (3) reveals the inherent coupling relationships between mechanistic side-wall root weld width *B_l_mec_*(*t*) and physical variables including initial groove gap width ${\tilde{G}}_{w}$, welding current *I* and arc voltage *U*, welding speed *V*, wire-feeding speed *V_f_*, and side-wall width ratio *κ* and layer height ratio χ. According to the UNG-AW experimental setup in this work, constant physical and empirical parameters are set as follows: wire radius *r* = 0.6 mm, specific enthalpy of weld metal *h_m_* = 853 kJ/kg, density *ρ* = 7.85 g/cm^3^, thermal-efficiency coefficient *η*_1_ = 0.85, and *η*_2_ = 1.8 denotes the correction coefficient for weld-layer height. The mechanism model is further globally calibrated using experimental welding samples, yielding an overall model correction coefficient *η*_3_ = 2.6.

This global correction coefficient *η*_3_ = 2.6 is calibrated solely on training-set and validation-set welding samples and kept fixed during subsequent neural-network training; test-set samples are not used for this mechanism-parameter fitting.

## 4. PI-Informer Multi-Step Prediction Model

To achieve high-precision and physically interpretable prediction of side-wall root weld width in ultra-narrow gap arc welding, this section proposes a physical constraint fusion mechanism based on the mechanism model in Equation (8) and constructs a PI-Informer short-term multi-step prediction model.

### 4.1. Architecture of the Standard Informer Model

**Figure 6:**
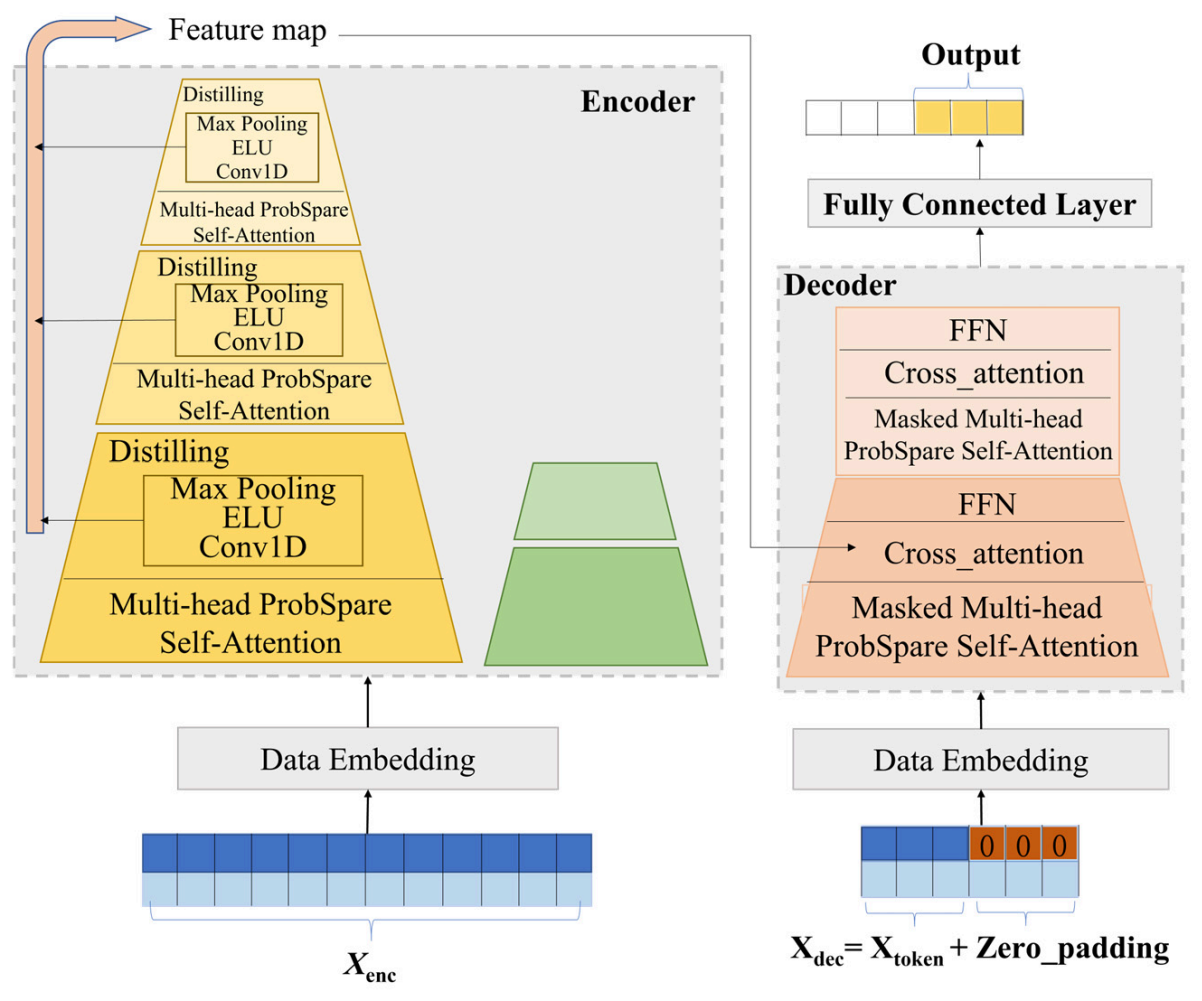
Structure framework of standard Informer model.

As a Transformer-derived model for long-sequence time-series forecasting, the Informer [18] is briefly reviewed in this subsection to clarify the baseline prior to introducing our welding-oriented modifications. It follows an encoder–decoder framework, as illustrated in Figure 6, consisting mainly of a data embedding layer, an encoder, a decoder, and a fully connected output layer. Herein, *X*_enc_, *X*_dec_, and *X*_token_ denote the encoder input, decoder input, and decoder start token, respectively. The data embedding layer generates high-dimensional representations for raw scalar time-series. Value embedding, positional encoding, and timestamp encoding are applied sequentially to produce three equal-dimension feature vectors, which are summed element-wise. This embedding strategy integrates numerical properties of input samples, relative positional cues, and physical temporal information into unified feature vectors.

Stacked attention units constitute the encoder to capture long-range temporal dependencies within input sequences of length *L*. Relying on the ProbSparse self-attention mechanism, the model lowers self-attention computational complexity from *O*(*L*^2^) to *O*(*LlogL*). Interleaved distillation layers utilize one-dimensional convolution, ELU activation, and max-pooling to progressively shorten sequence length, extract dominant temporal patterns, and mitigate memory overhead. Outputs from different distillation modules are concatenated to build multi-scale deep feature maps for subsequent decoding.

The decoder implements a generative multi-step forecasting paradigm, enabling all future predictions to be produced within one forward pass and thus improving inference efficiency. Its input is assembled by concatenating the tail portion of historical target sequence and zero-padded prediction placeholders, followed by projection into high-dimensional feature space via the data embedding layer. Each stacked decoder layer comprises masked multi-head ProbSparse self-attention, cross-attention, and feed-forward networks. Masked self-attention enforces temporal causality and models inner dependencies among tokens of the decoder input. In cross-attention, decoder-side features serve as Queries, while deep encoder outputs act as Keys and Values. This mechanism supports cross-scale information interaction between local transient patterns and global historical dependencies. The final fully connected layer projects aggregated features to obtain predicted time-series outputs.

### 4.2. Construction of the PI-Informer Model

**Figure 7:**
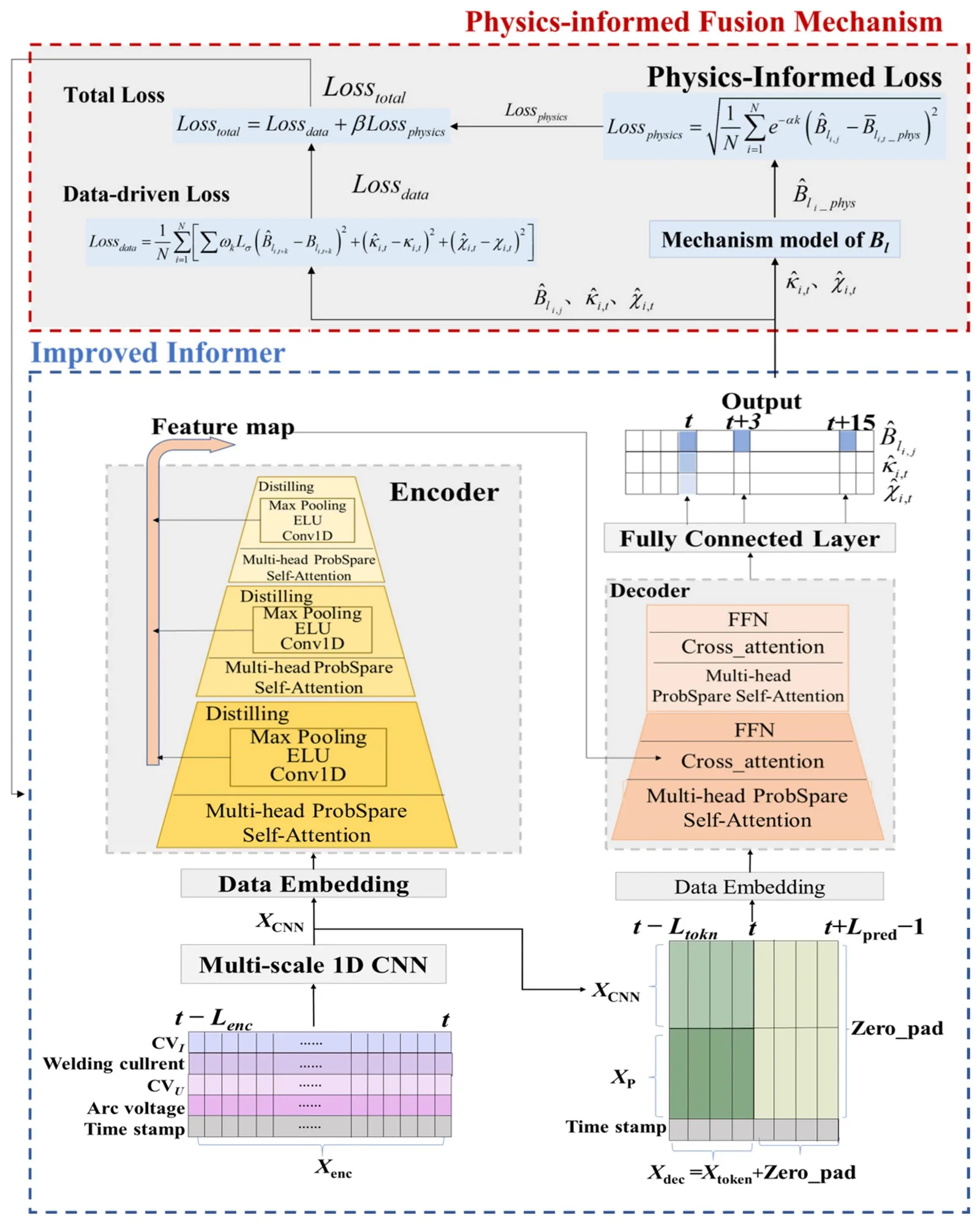
Architecture of PI-Informer model.

Targeting the time-series forecasting task for welding processes, improvements are implemented upon the standard Informer to construct the proposed PI-Informer prediction model. The overall architecture is illustrated in Figure 7. The model consists of a data-driven branch and a physics-constrained branch. *X*_enc_, *X*_dec_, *X*_token_, and *X*_mec_ denote the encoder input, decoder input, decoder token input, and mechanism-model input, respectively. *L*_enc_, *L*_token_, and *L*_pred_ represent the sequence length of *X*_enc_, the sequence length of *X*_token_, and the number of zero-padded placeholders, corresponding to the encoder historical window, decoder token window, and prediction sliding window, respectively.

The encoder input *X*_enc_ is a four-dimensional time-series vector concatenated from arc voltage U and its coefficient of variation CV_U_, welding current I and its coefficient of variation CV_I_ at each time step within the historical sliding window [*t* − *L*_enc_ – 1, *t*]. Unlike the standard Informer, which embeds raw sensor signals directly, a multi-scale 1D-CNN is introduced as the front-end feature extraction module to extract local transient electrical-signal features *X*_CNN_ from *X*_enc_, strengthening the model’s capability to capture local transient dynamics of welding electrical signals.

The standard Informer adopts the historical target sequence as the decoder start token. In this work, this mechanism is modified: the feature fragment *X*_CNN_token_ with length *L*_token_ is cropped from the tail of *X*_CNN_. It is then temporally aligned and linearly fused with a six-dimensional historical process-parameter vector *X*_p_, including pre-weld groove width *G*_w_, welding speed *V*, wire-feeding speed *V_f_*, arc voltage *U*, welding current *I*, and heat input *Q*, to form the decoder token *X*_token_. *X*_mec_ is a one-dimensional vector concatenated from pre-set process parameters *G*_w_, *V*, *V_f_*, together with arc voltage *U* and welding current *I* at the current time step *t*.

To further clarify the input configuration and inference workflow of the proposed PI-Informer, the forecasting information set and decoder implementation details are elaborated below.

The forecasting information set fed into the PI-Informer strictly consists of measurable variables available only at the current time instant *t*, without introducing any inaccessible future information.

The encoder input *X*_enc_ corresponds to multi-source time-series signals within the historical observation window [*t* − *L*_enc_ – 1, *t*], including arc voltage *U*, welding current *I*, and their corresponding coefficients of variation CV_U_ and CV_I_.

The mechanism-branch input *X*_mec_ is composed of fixed pre-weld process parameters: groove width *G*_w_, welding speed *V*, and wire-feed speed *V_f_*, together with instantaneously measured arc voltage *U* and welding current *I* obtained at time *t*.

The decoder start token *X*_token_ is generated by fusing deep features cropped from the tail output of multi-scale 1D-CNN and static process-parameter vector containing *G*_w_, *V*, *V_f_*, instantaneous *U*, *I*, and heat input *Q*. Since side-wall root weld width cannot be directly measured online in practical ultra-narrow-gap arc welding scenarios, the ground-truth target sequence adopted by vanilla Informer cannot be utilized as decoder start token in this task.

The complete decoder input *X*_dec_ is formed by concatenating the fused *X*_token_ with zero-padding placeholders reserved for future prediction horizons. No ground-truth labels of future time steps are fed into the decoder during inference.

The decoder adopts a non-autoregressive generative forecasting paradigm, which simultaneously produces multi-step predictions for horizons *t*, *t* + 3, and *t* + 15 within one single forward propagation pass. The decoder stack comprises masked multi-head ProbSparse self-attention, cross-attention and feed-forward networks. The masked multi-head ProbSparse self-attention enforces strict temporal causality and prevents information leakage from future time steps. In cross-attention operation, queries originate from decoder features, while keys and values are derived from deep latent features output by the encoder. Finally, a fully connected layer maps decoder high-dimensional features to prediction outputs, including side-wall root weld width at three horizons and auxiliary geometric parameters $\hat{\kappa }$ and $\hat{\chi }$.

#### 4.2.1. Data-Driven Branch

The data-driven branch performs time-series feature mining and multi-step parallel prediction. Its workflow is described as follows.

(1) The multi-scale 1D-CNN extracts instantaneous dynamic features from encoder inputs within the historical window [*t* − *L*_enc_ – 1, *t*]. After data embedding generates high-dimensional vectors integrating numerical, positional and temporal information, features are fed into stacked ProbSparse self-attention modules of the encoder to learn long-range temporal dependencies. Distillation layers further compress sequence length and extract dominant patterns. Multi-scale deep features, which combine local transient electrical-signal characteristics and global temporal dependencies from molten-pool heat accumulation, are finally obtained and transmitted to the cross-attention layer of the decoder.
(2) The decoder input is projected into high-dimensional embedding vectors via the data embedding layer. Unmasked self-attention learns dependencies between transient electrical-signal patterns and welding process parameters. Hierarchical cross-attention is subsequently applied, where decoder-side features serve as Queries and multi-scale deep encoder outputs act as Keys and Values. Information fusion is achieved among welding process parameters, long-term molten-pool thermal dynamics, and short-term dynamic responses under arc heating.
(3) To mitigate error accumulation induced by autoregressive iteration, non-autoregressive parallel decoding is adopted for multi-step forecasting. The fused decoder features are mapped to the target output space through the fully connected layer. Within a single forward propagation, the model outputs the predicted side-wall root weld width ${\hat{B}}_{l_{i,t}}$, ${\hat{B}}_{l_{i,t+3}}$, and ${\hat{B}}_{l_{i,t+15}}$ at *t*, *t* + 3, *t* + 15 as well as the predicted mechanism parameters ${\hat{\kappa }}_{i,t}$ and ${\hat{\chi }}_{i,t}$ at time *t* for the *i*-th sample within the prediction sliding window [*t*, *t* + *L*_pred_ − 1].

#### 4.2.2. Physics-Constrained Branch

The physics-constrained mechanism complies with causal priors for time-series forecasting. Each loss term is normalized before weighted summation to unify their numerical scales. The implementation procedure of physics-constrained fusion is given below:

(1) The data-driven loss is defined as
  (9) $${\mathrm{Loss}}_{\mathrm{data}}=\frac{1}{N}\sum_{i=1}^{N}\left[\sum_{k}\omega_{k}L_{\sigma }\left(\frac{{\hat{B}}_{l_{i,t+k}}-B_{l_{i,t+k}}}{B_{l_{\mathrm{ref}}}}\right)+{\left(\frac{{\hat{\kappa }}_{i,t}-\kappa_{i,t}}{\kappa_{\mathrm{ref}}}\right)}^{2}+{\left(\frac{{\hat{\chi }}_{i,t}-\chi_{i,t}}{\chi_{\mathrm{ref}}}\right)}^{2}\right]$$
  where *N* denotes the batch size and *ω_k_* represents the loss-weight coefficient; the time step *k* is {0,3,15}. Prediction errors of side-wall root weld width are calculated only at three forecast moments *t*, *t* + 3, and *t* + 15, whereas prediction errors for unknown mechanism-model parameters are computed solely at time t. Remaining time steps are excluded from loss computation. *κ*_ref_ and *χ*_ref_ denote the statistical mean values of the side-wall fusion width ratio *κ* and the layer height ratio *χ* over the training set; the relevant data are listed in Table 1. The reference $B_{l_{\mathrm{ref}}}$ is calculated by the mechanistic model, using the parameters provided in Table 1.

A weighted Huber loss is employed for these three critical moments:

(10) $$L_{\sigma }\left({\hat{B}}_{l_{i,t+k}}-B_{l_{i,t+k}}\right)=\left\{\begin{array}{cc} \frac{1}{2}{\left({\hat{B}}_{l_{i,t+k}}-B_{l_{i,t+k}}\right)}^{2}, & \;\;\;\;\;\left|{\hat{B}}_{l_{i,t+k}}-B_{l_{i,t+k}}\right|\leq \sigma \\ \sigma \left(\left|{\hat{B}}_{l_{i,t+k}}-B_{l_{i,t+k}}\right|-\frac{1}{2}\sigma \right), & \;\;\;\;\;\mathrm{others} \end{array}\right.$$

where σ is the segmentation threshold of Huber loss, set as σ = 1.0 mm in this study. Squared loss is used within the small-error region to guarantee convergence accuracy, while linear loss is adopted for large errors to suppress interference from outlier samples. Considering practical requirements of intelligent welding control, different forecast horizons possess distinct engineering significance and are assigned differentiated loss weights. Time instant *t* supports current-state monitoring; *t* + 3 supports short-term trend judgement with insufficient lead time for effective control decisions; *t* + 15 represents a relatively long prediction horizon investigated in this multi-step forecasting task and is assigned the highest loss weight for forecasting performance optimization. Corresponding loss weights for each forecast horizon are set as *ω*_0_ = 1.0, *ω*_3_ = 1.3, *ω*_15_ = 2.0 accordingly.

(2) The predicted mechanism parameters ${\hat{\kappa }}_{i,t}$, ${\hat{\chi }}_{i,t}$ together with input *X*_mec_ are substituted into the mechanism model given in Equation (8) to solve for the physically expected side-wall root weld width ${\bar{B}}_{l_{i,t\_\mathrm{phys}}}$ at the starting time instant t of the prediction sliding window.
(3) In this study, *k* ∈ {0,3,15} denotes the prediction lead steps corresponding to horizons *t*, *t* + 3 and *t* + 15, respectively. ${\bar{B}}_{l_{i,t\_\mathrm{phys}}}$ represents the side-wall root fusion width calculated by the static mechanistic model, which is computed solely from measurable process parameters available at the current time instant t. The mechanistic model cannot be forward-simulated to generate physical references for future instants *t* + *k*, since the arc electrical signals at future moments are unavailable during real multi-step prediction. To alleviate the risk of penalizing genuine physical evolution over long prediction horizons, an exponential time-decay factor *e*^−*αk*^ is introduced, which reduces the weight of physical regularization as the prediction horizon increases. The implemented physics-constraint loss is formulated as
  (11) $${\mathrm{Loss}}_{\mathrm{physics}}=\sqrt{\frac{1}{N}\sum_{i=1}^{N}\sum_{k\in \{0,3,15\}}e^{-\mathrm{\alpha k}}\cdot {\left({\hat{B}}_{l_{i,t+k}}-{\bar{B}}_{l_{i,t}\_\mathrm{phys}}\right)}^{2}}$$
  where *α* denotes the decay coefficient, which adjusts how rapidly physical-constraint strength decays with increasing prediction horizon. Physical constraints are activated only for forecast horizons *t*, *t* + 3, and *t* + 15. The physical-constraint weight reaches its maximum at *k* = 0 (time instant *t*) and decays exponentially as *k* increases, which gradually weakens the physical constraint effect.
(4) The total loss function *Loss_total_* of the PI-Informer model is obtained by weighted summation of data-driven *Loss_data_* and physics-constrained loss *Loss_physics_*
  *Loss_total_* = *Loss_data_* + *βLoss_physics_* (12)
  where *β* is the weight coefficient of physical constraints, which regulates physical-constraint intensity during model training.
(5) The PI-Informer network is optimized by minimizing the total loss *Loss_total_*.

### 4.3. Short-Term Multi-Step Prediction Method Based on PI-Informer

In this work, a multi-step prediction approach for side-wall root weld width is established based on the PI-Informer, which balances prediction accuracy and physical consistency. The whole procedure consists of two stages: model training and online prediction. In the offline training stage, the model is trained on historical datasets to obtain optimal network parameters. In the online prediction stage, real-time acquired data are fed into the trained model to realize online prediction during the welding process.

#### 4.3.1. Offline Model Training

**Figure 8:**
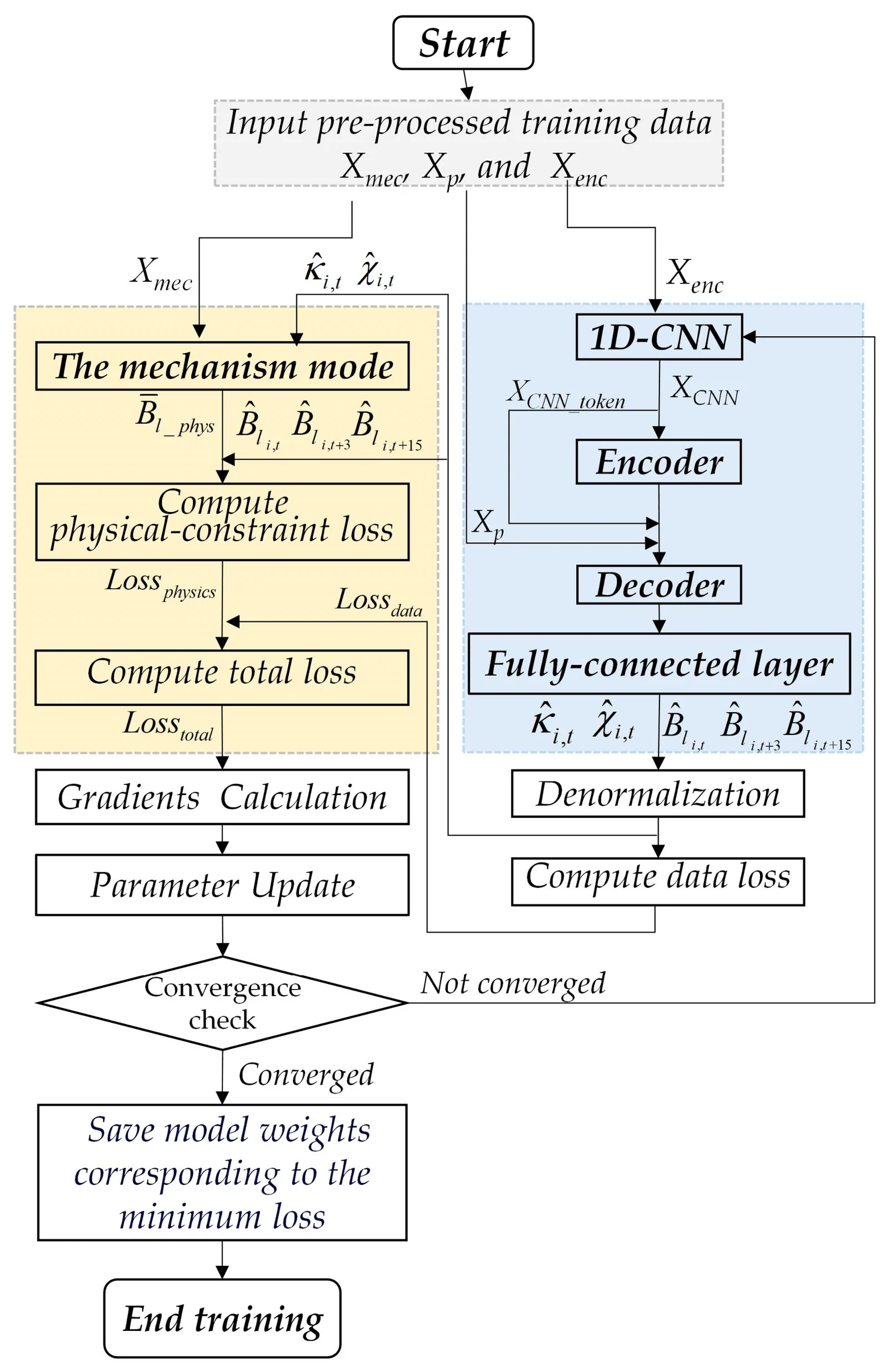
Training workflow of the PI-Informer model.

Offline training is carried out by minimizing the total loss function *Loss_total_*, and the training workflow is illustrated in Figure 8. This subsection mainly describes the forward propagation, physical-constraint embedding, and parameter-updating mechanism of PI-Informer. Detailed data preprocessing procedures are provided in Section 5.1.

The specific steps are summarized as follows:

(1) Preprocessed training samples are fed into the PI-Informer for forward propagation. The multi-scale 1D-CNN extracts instantaneous dynamic features *X*_CNN_ from the encoder input *X*_enc_. These features are further processed by the encoder to learn long-range multi-scale deep features, which are then delivered to the decoder.
(2) Combined with the process-parameter vector *X*_p_, feature sequence *X*_CNN_token_ and *X*_CNN_, the decoder outputs the predicted side-wall root weld-width values ${\hat{B}}_{l_{i,t}}$, ${\hat{B}}_{l_{i,t+3}}$, and ${\hat{B}}_{l_{i,t+15}}$ at three forecast horizons *t*, *t* + 3, and *t* + 15 within the prediction sliding window [*t*, *t* + *L*_pred_−1], together with the predicted mechanism parameters ${\hat{\kappa }}_{i,t}$ and ${\hat{\chi }}_{i,t}$ at time *t,* in a single forward pass.
(3) The denormalized predicted mechanism parameters ${\hat{\kappa }}_{i,t}$, ${\hat{\chi }}_{i,t}$ and *X*_mec_ are substituted into the mechanism model in Equation (8) to calculate the physically expected side-wall root weld width at time *t*.
(4) Calculation of the total loss *Loss_total_*. The denormalized predicted side-wall root weld-width values and predicted mechanism parameters are substituted into Equation (9) to compute the data-driven loss *Loss_data_*. Meanwhile, the predicted side-wall root weld-width values and the corresponding physical expectation are substituted into Equation (11) to obtain the physics-constrained loss *Loss_physics_*. Finally, *Loss_data_* and *Loss_physics_* are combined according to Equation (12) to yield the total loss *Loss_total_* of the PI-Informer model.
(5) Backward propagation and parameter update. Gradients for all trainable parameters in the Informer network are computed according to the total loss *Loss_total_*, and the Adam optimizer is employed to update network weights. The mechanism-model parameters *G_w_*, *V*, *V_f_*, *h_m_*, *ρ*, and *r* are excluded from gradient updating.
(6) Convergence judgement. The validation-set loss is calculated after each training epoch. Early-stopping is triggered to terminate training and save the model weights with the minimum validation-set loss if the validation loss stops decreasing for 50 consecutive epochs. If neither the early-stopping criterion nor the maximum epoch number is reached, the procedure returns to Step (1) for further iterations.

#### 4.3.2. Short-Term Multi-Step Online Prediction of Side-Wall Root Weld Width

**Figure 9:**
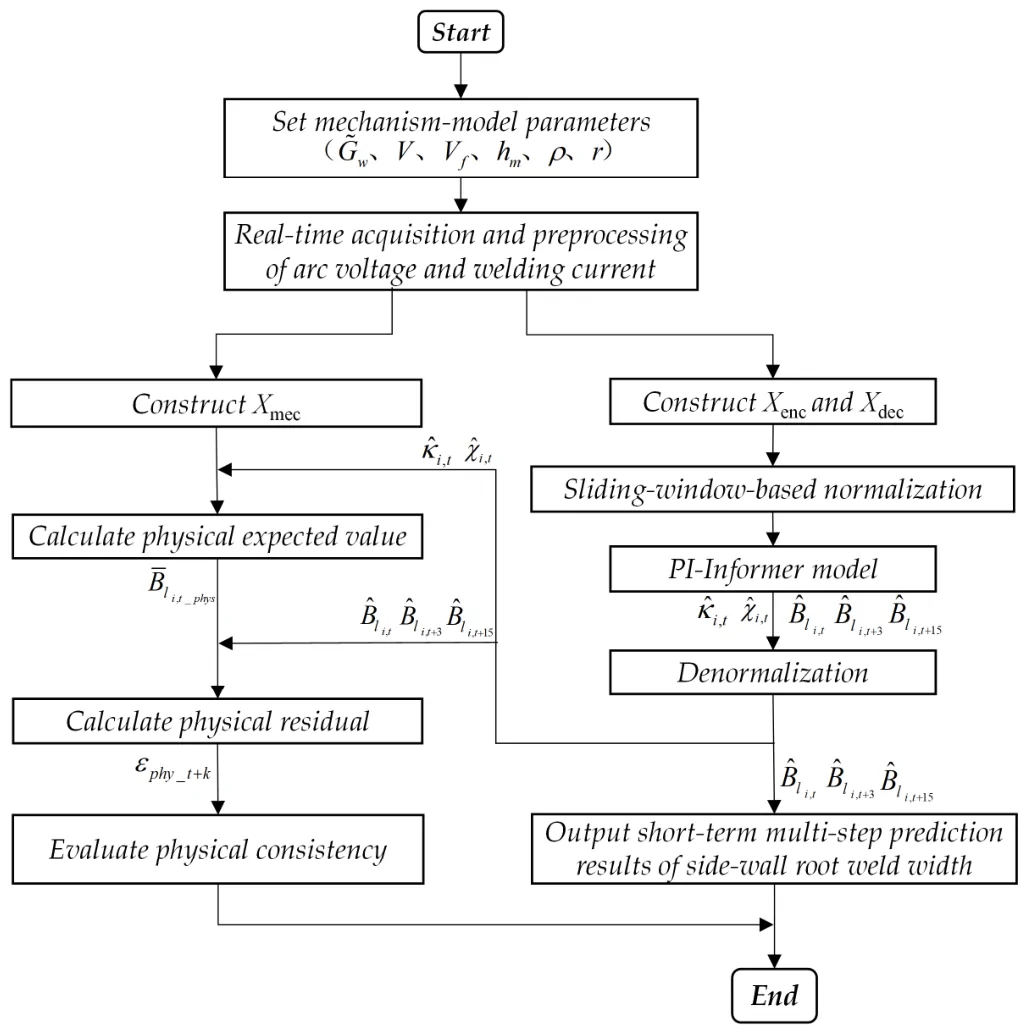
Principle of the short-term multi-step prediction method for *B_l_*.

The trained PI-Informer model is applied to the ultra-narrow-gap arc welding process to realize short-term multi-step online prediction, and the prediction workflow is presented in Figure 9. First, the real-time collected arc voltage and welding-current signals are resampled and filtered, and the mechanism-model parameters *G_w_*, *V*, *V_f_*, *h_m_*, *ρ*, and *r* are initialized according to the pre-welding process parameters. Second, the encoder input *X*_enc_ and decoder input *X*_dec_ are constructed, normalized within the sliding window, and fed into the well-trained PI-Informer. Through forward propagation, the model outputs the predicted side-wall root weld-width values ${\hat{B}}_{l_{i,t}}$, ${\hat{B}}_{l_{i,t+3}}$, and ${\hat{B}}_{l_{i,t+15}}$ for three forecast horizons, as well as the predicted mechanism parameters ${\hat{\kappa }}_{i,t}$ and ${\hat{\chi }}_{i,t}$ at time instant *t*, in a single forward pass. The prediction outputs are denormalized to obtain multi-step prediction results of side-wall root weld width, which reflect its short-term variation trend. Meanwhile, the physically expected value ${\bar{B}}_{l_{i,t\_\mathrm{phys}}}$ is calculated from the mechanism model. Substituting this physical expectation and multi-step prediction results into Equation (13), the physical residual *ε_phy_*__*t*+*k*_ at each prediction horizon is computed. *ε_phy_*__*t*+*k*_ is an auxiliary index for quantifying the deviation between model prediction and the static mechanistic baseline, and it is integrated into the composite loss to impose physical constraints during training. It should be emphasized that *ε_phy_*__*t*+*k*_ serves only as a soft regularization term during model training rather than an independent indicator for verifying physical rationality. The primary evidence for evaluating prediction reliability is the metallographic ground-truth of side-wall root fusion width.

(13) $$\varepsilon_{\mathrm{phy}\_t+k}=\left|{\hat{B}}_{l_{i,t+k}}-{\bar{B}}_{l_{i,t}\_\mathrm{phys}}\right|$$

## 5. Experimental Results and Discussion

### 5.1. Data Preprocessing, Dataset Construction and Evaluation Metrics

To verify the effectiveness of the proposed method, straight-groove workpieces with varying groove widths ranging from 4.9 mm to 6.2 mm were manufactured on an ultra-narrow-gap arc welding experimental platform. Multiple groups of ultra-narrow-gap arc welding trials were performed with varied welding voltages, wire-feed speeds, and welding speeds. Representative valid process–parameter combinations are summarized in Table 2. Some trials suffered from unstable arcs and assembly-related defects and were thus excluded from the final dataset.

#### 5.1.1. Data Preprocessing

To eliminate sensor noise interference and match sampled data with the physical process of droplet metal transfer, resampling and filtering were performed on raw arc voltage and welding-current data using the droplet transfer period as the sampling time step. In ultra-narrow-gap arc welding, globular transfer serves as the dominant metal-transfer mode under stable welding conditions, with a typical globular-transfer period of approximately 50 ms. In this study, the average value of every consecutive 2500 raw sampling points was calculated and treated as one new sampling point. Each obtained sampling point corresponds to the complete procedure from droplet formation to its transfer into the molten pool, and the average arc voltage and welding current for each droplet were accordingly acquired. Furthermore, to enhance the model’s perception capability for arc stability, the coefficient of variation in arc voltage CV_U_ and coefficient of variation in welding current CV_I_ were computed within the sliding window as implicit dynamic features of arc stability for subsequent extraction by the convolutional module.

#### 5.1.2. Dataset Construction

A sliding-window mechanism was adopted to convert continuous time-series signals into learning samples. The side-wall root formation is mainly governed by molten-pool heat accumulation and thermal effects under effective arc constraint. The heat-accumulation duration of the molten pool depends on molten-pool length and welding speed, while the arc thermal-action duration is determined by the thermal-interaction range of the arc along the welding direction. Taking a groove width of 5 mm as an example, under optimal welding parameters with a welding speed of 10 mm/s, the typical molten-pool length ranges from 20 mm to 23 mm, and the typical arc thermal-action range constrained by flux slices along the welding direction is approximately 6 mm. Consequently, the molten-pool heat-accumulation time reaches 2–2.3 s, and the arc thermal-action time is around 600 ms. To enable the model to fully learn molten-pool heat-accumulation characteristics and arc heat-input behaviours, the historical window length parameters were set as *L*_enc_ = 40, *L*_token_ = 12, and *L*_pred_ = 16. Under this configuration, the model exploits historical data over the past 2 s to sufficiently capture long-term temporal dependencies of electrical signals during molten-pool heat accumulation. Based on electrical-signal features within the most recent 600 ms together with process-parameter vectors, the model obtains recent arc behaviours and further predicts side-wall root weld width at three forecast horizons *t*, *t* + 3, *t* + 15 within the future 800 ms window, as well as unknown mechanism parameters at instant *t*, so as to characterize short-term variation trends.

#### 5.1.3. Dataset Partition and Normalization

Datasets were partitioned on a weld-bead basis. Specifically, 70%, 10%, and 20% of the total weld beads were allocated to the training, validation, and test sets, respectively, which fully preserves the inherent temporal continuity of sequential data within each weld bead. The training set was responsible for network parameter optimization, while the validation set was used for early-stopping criteria determination and hyperparameter optimization. The test set remained completely unseen throughout the entire training and tuning process and was only employed to evaluate the generalized prediction performance of the final optimized model. Considering the scale sensitivity of the Informer model, sliding-window Min–Max normalization was performed to normalize all input features into the range [0, 1]. This preprocessing eliminates dimensional disparities and accommodates local amplitude variations in welding electrical signals. After rigorous data screening and noise cleaning, a total of 1200 valid samples were established for model training and evaluation.

#### 5.1.4. Evaluation Metrics

For each prediction step *k* ∈ {0,3,15}, the root-mean-square error (RMSE), mean absolute error (MAE), mean absolute percentage error (MAPE), and batch-averaged physical residual ${\bar{\varepsilon }}_{\mathrm{phy}}$ are calculated independently. These four metrics are adopted to comprehensively evaluate the model performance for the multi-step prediction task. RMSE and MAE quantify the deviations between predicted and measured values. Specifically, RMSE is more sensitive to large prediction errors, while MAE reflects the actual physical magnitude of errors. MAPE describes the average percentage deviation in predictions from ground-truth values and provides a dimensionless measure of relative error. Smaller values of these three metrics correspond to higher model prediction accuracy. The batch-averaged physical residual ${\bar{\varepsilon }}_{\mathrm{phy}}$ quantifies the average discrepancy between model predictions and their corresponding physical-expectation values for samples within each batch. A smaller batch-averaged physical residual ${\bar{\varepsilon }}_{\mathrm{phy}}$ indicates improved agreement between predictions and the mechanism-model expectation, serving as an auxiliary indicator of physical consistency.

(14) $${\mathrm{RMSE}}_{t+k}=\sqrt{\frac{1}{N}\sum_{i=1}^{N}{\left({\hat{B}}_{l_{i,t+k}}-B_{l_{i,t+k}}\right)}^{2}}$$

(15) $${\mathrm{MAE}}_{t+k}=\frac{1}{N}\sum_{i=1}^{N}\left|{\hat{B}}_{l_{i,t+k}}-B_{l_{i,t+k}}\right|$$

(16) $${\mathrm{MAPE}}_{t+k}=\frac{100}{N}\sum_{i=1}^{N}\left|\frac{{\hat{B}}_{l_{i,t+k}}-B_{l_{i,t+k}}}{B_{l_{i,t+k}}}\right|$$

(17) $${\bar{\varepsilon }}_{\mathrm{phy}\_t+k}=\sqrt{\frac{1}{N}\sum_{i=1}^{N}{\left({\hat{B}}_{l_{i,t+k}}-{\bar{B}}_{l_{i}\_\mathrm{phys}}\right)}^{2}}$$

### 5.2. Experimental Environment and Parameter Settings

The hardware configuration for the proposed model, comparative experiments and ablation experiments consisted of an Intel Core i9-12900K CPU (Intel Corporation, Santa Clara, CA, USA), 64 GB RAM, and an NVIDIA T1200 GPU with 14 GB video memory (NVIDIA, Santa Clara, CA, USA). The software environment was built with Python 3.10, PyTorch 2.51 and CUDA 12.6.

To construct a lightweight model balancing prediction accuracy and inference efficiency, the number of encoder layers was set to 3, and the decoder layer count was set to 2. The number of heads for ProbSparse self-attention was 8, and the embedding dimension was set to 256. The ELU function was selected as the activation function. Four parallel convolution kernels of sizes 3, 5, 7, and 9 were adopted for the multi-scale 1D-CNN module. The physically constrained loss function proposed in this work was utilized as the objective loss. The Adam optimizer was applied for model training with a maximum epoch number of 200, and an early-stopping strategy was introduced to mitigate overfitting. Furthermore, the Particle Swarm Optimization (PSO) algorithm was used for hyperparameter searching over learning rate, batch size and decay coefficient *α*.

Table 3 shows the PSO-optimized hyperparameter configurations for the comparative models. All competing models were individually tuned via particle swarm optimization. Training-related hyperparameters (learning rate and batch size) shared identical search ranges for all models. Architecture-specific hyperparameters adopted model-appropriate search spaces according to each network’s intrinsic structure. Only the maximum training epoch (200), Adam optimizer, and early-stopping patience (50) were kept identical across all models to guarantee fair comparison. Using the dataset consisting of 1200 valid time-series samples, the average training wall-clock time for one complete training run (until convergence by early-stopping or maximum epoch) for each competing model is listed as: LSTM 8.2 min, GRU 7.6 min, CNN-LSTM 10.4 min, TCN 10.4 min, vanilla Informer 13.1 min, Transformer 15.3 min, and PI-Informer 16.7 min. The extra computation cost of PI-Informer originates from multi-scale 1D-CNN feature extraction and physical-residual loss calculation in the physics-constrained branch. All PSO hyperparameter searches were performed in serial, and the total wall-clock time for independent PSO searching across all competing models reaches 21.4 h.

### 5.3. Ablation Experiments

Ablation experiments are carried out to quantify the individual contributions of the physical-constraint module and the 1D-CNN front-end feature extraction module within the PI-Informer for multi-step prediction of side-wall root weld width. Each group of experiments is independently trained and tested five times, and the final model performance is reported as the mean value together with the standard deviation calculated from the five replicated runs.

#### 5.3.1. Ablation Experiments on Physical Constraint

The strength of the physical constraint is controlled by the weight coefficient *β*. To preserve the dominant role of data-driven learning, the physical constraint adopted in this work serves as a soft regularizer rather than a hard-constraint term. With the complete model architecture kept unchanged, ablation experiments are carried out for *β* ∈ {0.00,0.01,0.02,0.04,0.045,0.05,0.055,0.10}. When *β* = 0.00, the model degenerates into the purely data-driven CNN-Informer model.

Table 4 summarizes the prediction errors at the three forecast horizons under different physical-constraint weights, where the values following the symbol ± denote the standard deviations obtained from five repeated experiments.

As illustrated in Table 2, prediction accuracy and physical consistency exhibit an initial improvement followed by degradation as *β* increases. When *β* rises from 0.00 to 0.05, both prediction accuracy and physical consistency are enhanced across all three forecast horizons. Specifically, the MAE at *t* + 3 decreases by 20.14%, and the physical residual at the long-range horizon *t* + 15 achieves the most prominent reduction of 36.79%. These results verify the effectiveness of the introduced physical constraint for multi-step prediction, especially its remarkable improvement in physical consistency for long-horizon forecasts. Further increasing *β* to 0.10 yields an obvious rise in all error metrics, which indicates that excessively strong physical constraints suppress data-driven model learning. The optimal physical consistency is achieved at *β =* 0.05, and a larger *β* beyond this point leads to simultaneous deterioration of prediction accuracy and physical consistency. Accordingly, *β* = 0.05 is selected as the optimal trade-off value. Under this configuration, sufficient physical guidance is provided without hindering the learning of data-driven temporal patterns, and the best balance between prediction accuracy and physical consistency is attained for all three forecast horizons.

**Figure 10:**
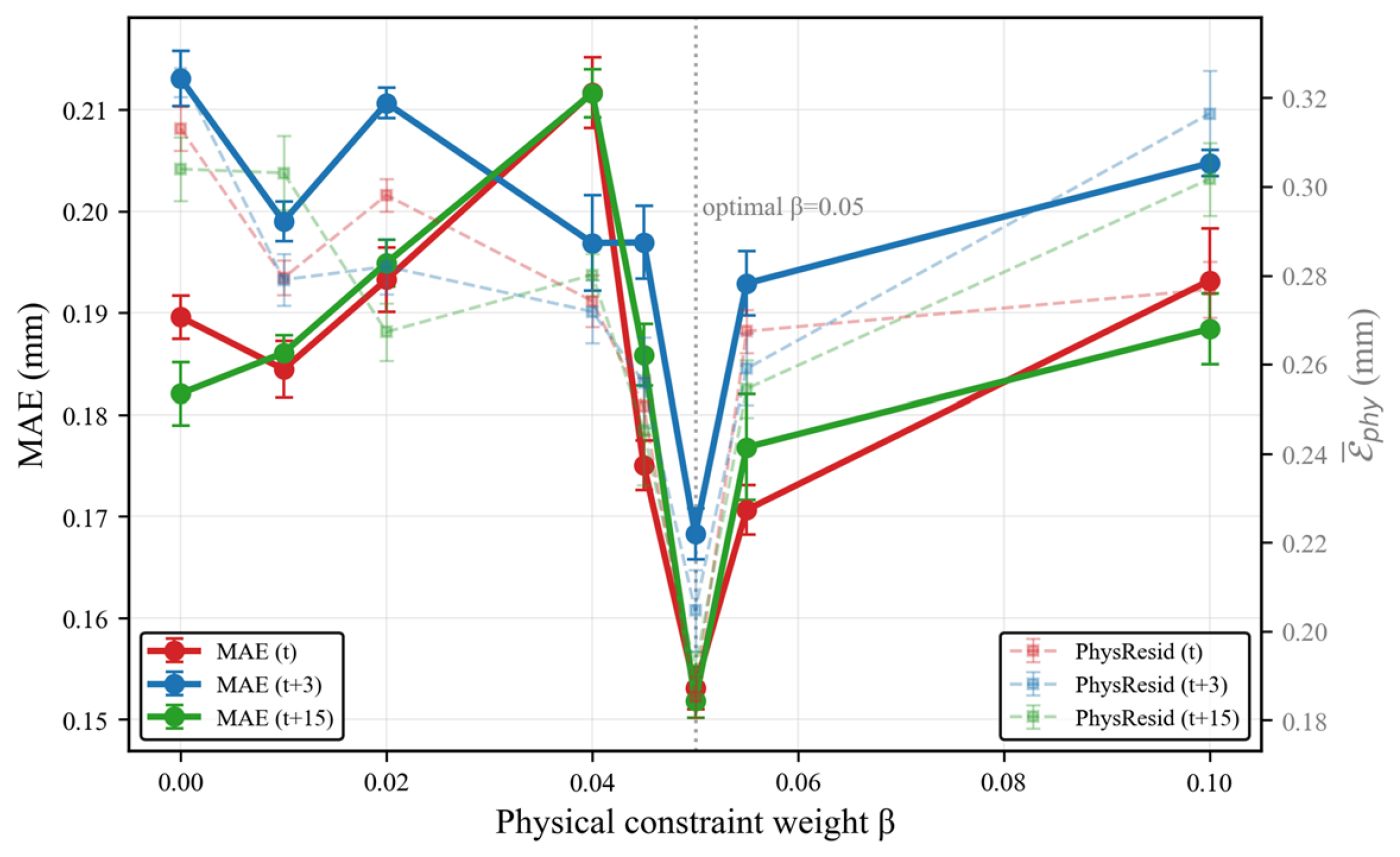
Sensitivity curve for the parameter *β*.

Figure 10 presents line plots of MAE and batch-averaged physical residual ${\bar{\varepsilon }}_{\mathrm{phy}}$ of PI-Informer under varying physical-constraint weight *β*. Each plotted result corresponds to the mean ± standard deviation in five independent experiments, and error bars in the figure reflect the dispersion of experimental outputs. As *β* increases from 0.00 to 0.05, the MAE values for the three prediction horizons *t*, *t* + 3 and *t* + 15 generally decline and reach their minima at *β* = 0.05. This demonstrates that moderate physical constraint effectively regularizes the training procedure and improves prediction accuracy while enhancing physical consistency. The shorter error bars at *β* = 0.05 also reveal superior training stability across repeated runs. When *β* is further increased to 0.10, MAE increases evidently, implying that overly heavy physical constraints excessively suppress the learning of data-driven features and weaken the model’s capability to fit local transient variations. Meanwhile, the batch-averaged physical residual generally decreases with enhanced physical constraint at small *β* and reaches a low level near *β* = 0.05, suggesting that appropriate physical constraints help improve the physical consistency of predictions. Nevertheless, further raising *β* to 0.10 causes the physical residual to rebound. This phenomenon shows that overly strong constraints cannot continuously reduce physical residuals but instead break the balance between data loss and physical loss. Combining the two metrics of MAE and batch-averaged physical residual ${\bar{\varepsilon }}_{\mathrm{phy}}$, *β* = 0.05 achieves a favourable trade-off among prediction accuracy, physical consistency and training stability. Combining the two metrics of MAE and batch-averaged physical residual ${\bar{\varepsilon }}_{\mathrm{phy}}$ obtained from validation-set beads, *β* = 0.05 achieves a favourable trade-off among prediction accuracy, physical consistency and training stability. The test-set results in Table 4 are reported after this hyperparameter was fixed, which is useful only for final performance evaluation rather than hyperparameter tuning.

#### 5.3.2. Ablation Experiment for the 1D-CNN Module

With all other experimental conditions unchanged and the optimal physical-constraint weight adopted, ablation experiments were performed by enabling and removing the 1D-CNN module. The prediction performances at the three forecast horizons *t*, *t* + 3, and *t* + 15 are summarized in Table 5, where w/ CNN denotes the complete model equipped with the 1D-CNN module, and w/o CNN represents the baseline model without the CNN front-end.

As shown in Table 5, the model integrated with the 1D-CNN module outperforms the baseline counterpart across all forecast horizons in terms of all evaluation metrics. At the current time step *t*, the RMSE of the w/-CNN model is reduced by 28.26% relative to the w/o-CNN baseline, accompanied by considerable decreases in MAE, MAPE and batch-averaged physical residual ${\bar{\varepsilon }}_{\mathrm{phy}}$. At the forecast horizon *t* + 3, the relative reduction in RMSE reaches 18.36%, and physical consistency is also improved. At *t* + 15, the relative performance gain originating from the CNN module declines moderately; nevertheless, PI-Informer (w/CNN) still achieves higher prediction accuracy and lower physical error. Overall, the contribution of local transient features gradually weakens as the prediction step increases, yet the 1D-CNN module consistently yields positive performance gains. In addition, smaller standard deviations are observed for repeated experiments under the w/-CNN configuration, demonstrating that the multi-scale 1D-CNN not only improves prediction accuracy and physical consistency but also facilitates stable model convergence.

**Figure 11:**
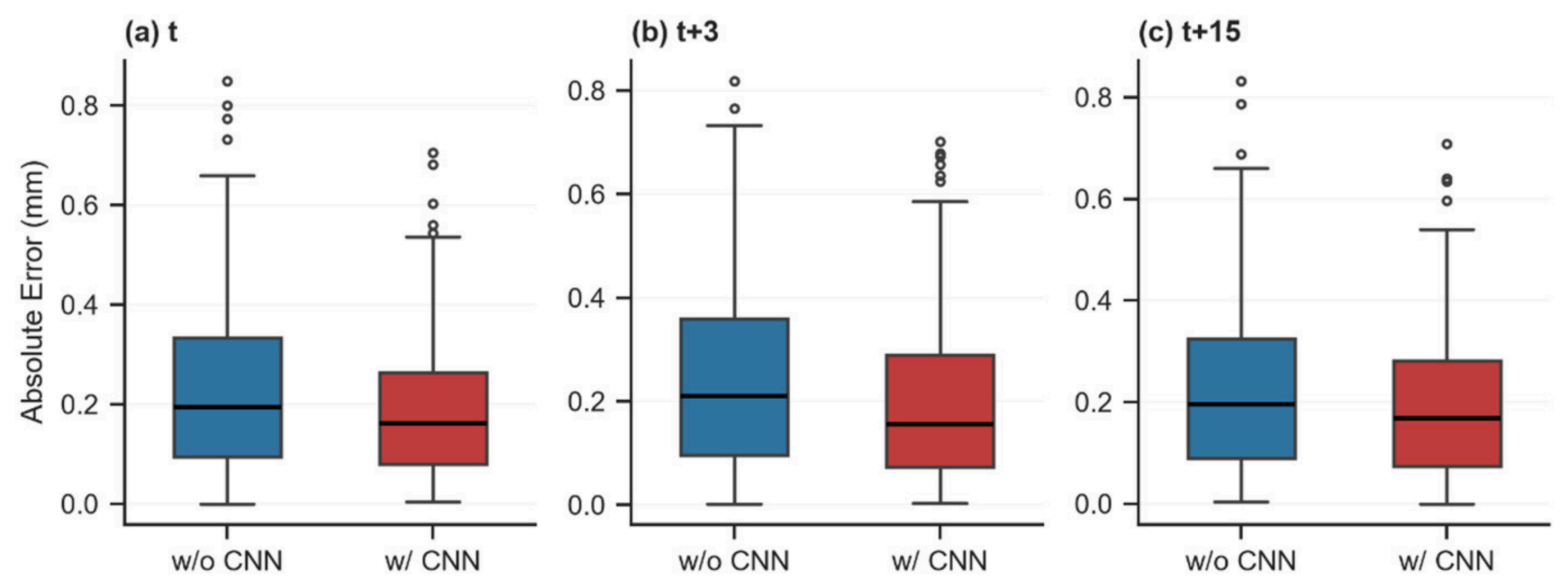
Comparison of errors with and without CNN. (**a**) Prediction horizon t; (**b**) prediction horizon \(t + 3\); (**c**) prediction horizon \(t + 15\).

Figure 11 shows the boxplots of absolute prediction errors on the test set from five repeated experiments, where each box reflects the error distribution across test samples. It can be seen that the model w/CNN yields generally lower absolute errors than the w/o CNN baseline across all prediction horizons, with the most prominent reduction observed at horizon *t*, demonstrating that the multi-scale 1D-CNN achieves the strongest suppression of short-term prediction error. Although outliers with large absolute errors appear above the boxes at *t*, *t* + 3 and *t* + 15, the main box bodies and medians of the two configurations show little overlap, and the error distribution of w/CNN consistently lies in a lower-error region, which verifies that the performance improvement is stable and consistent rather than a random fluctuation. The overall downward shift and narrower interquartile range of the error distribution indicate that the 1D-CNN module not only reduces the average prediction error but also compresses error dispersion and mitigates large deviations, enabling the model to achieve better prediction robustness at all three horizons of *t*, *t* + 3 and *t* + 15.

**Figure 12:**
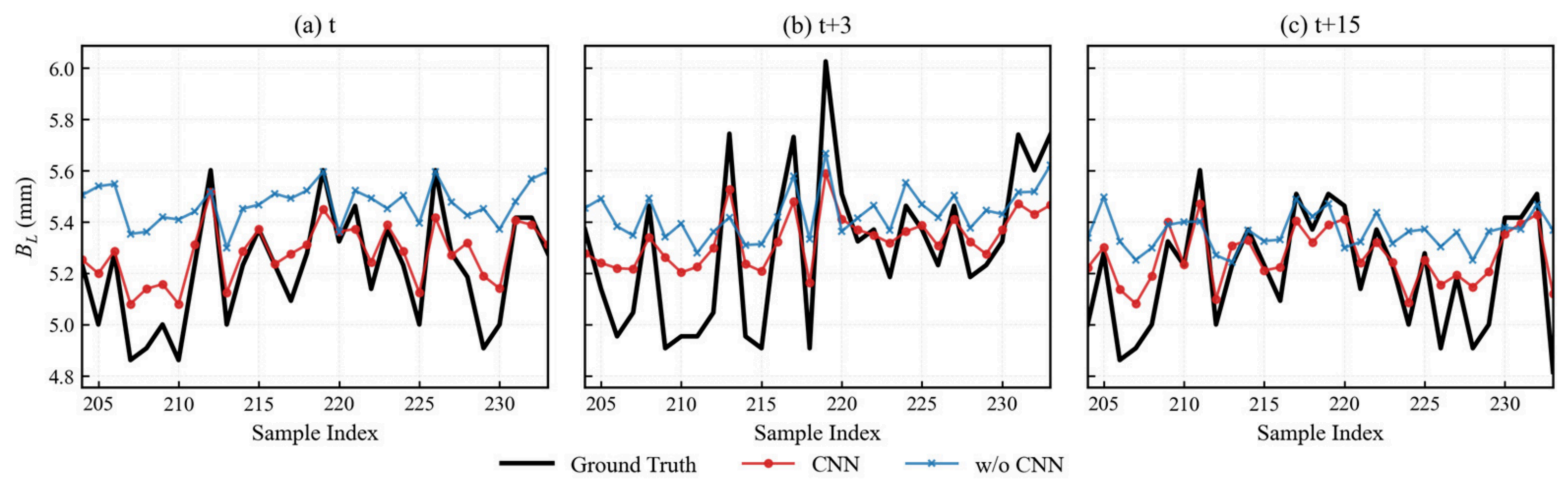
Comparison of prediction curves for CNN ablation experiment. (**a**) Prediction horizon t; (**b**) prediction horizon \(t + 3\); (**c**) prediction horizon \(t + 15\).

Figure 12 compares prediction curves of a representative weld bead. The black solid line corresponds to ground-truth values, the blue solid line denotes the w/o-CNN baseline model, and the red solid line represents the full model with the 1D-CNN module. It is evident that the w/-CNN model can track rapid fluctuations and local abrupt variations more precisely, whereas the w/o-CNN model exhibits obvious prediction lag and over-smoothing artefacts. Instantaneous dynamic information such as droplet transfer and arc morphology is embedded within welding electrical signals. The w/o-CNN model solely relies on the global self-attention mechanism of the vanilla Informer encoder for feature extraction. Owing to the intrinsic cross-sequence weighted-averaging operation, it tends to capture slow long-range trends of electrical signals while discarding abundant transient details, which further leads to prediction lag and smoothing distortion when confronting rapid oscillations of welding signals. In contrast, the w/-CNN model employs parallel multi-scale 1D-CNN kernels of sizes 3, 5, 7, and 9 to mine local temporal patterns. Small convolution kernels capture instantaneous variations between adjacent sampling points and preserve short-term dynamic information such as droplet transfer, while large kernels cover longer time windows and characterize long-term evolutionary trends induced by molten-pool heat accumulation. This design compensates for the deficiency in Informer global attention in modelling local transient features.

In summary, the prediction trajectories of the w/-CNN model fit the ground-truth side-wall root weld width more closely at all forecast horizons of *t*, *t* + 3 and *t* + 15. This demonstrates that the proposed module enhances the tracking capability for instantaneous dynamic mutations in single-step prediction and provides sufficient local feature support for multi-horizon prediction.

### 5.4. Comparative Experiments and Result Analysis

Prior to comparative experiments, particle swarm optimization (PSO) was executed independently for each competing model. Every model acquired its own optimal combination of hyperparameters. Training-related hyperparameters (learning rate, batch size and key architectural hyperparameters) adopted identical search ranges for all models, whereas architecture-specific hyperparameters used model-appropriate search spaces matching each network’s intrinsic structural characteristics. For fair comparison, only the maximum training epoch, Adam optimizer type and early-stopping patience were maintained identically for all models. Detailed PSO-optimised hyperparameter values for each model are summarized in Section 5.2 as shown in Table 2.

To validate the performance of the proposed PI-Informer for short-term multi-step prediction of side-wall root weld width, comparative experiments were conducted against three mainstream time-series models including LSTM, GRU, and standard Informer. To guarantee fair comparison, identical settings of training epochs, batch size and optimizer were adopted for all deep learning models. RMSE, MAE, MAPE and the batch-averaged physical residual ${\bar{\varepsilon }}_{\mathrm{phy}}$ were employed as unified evaluation metrics. To eliminate accidental effects caused by random weight initialization, optimizer stochasticity and data-loading order, and to verify the statistical stability and generalization capability of the proposed approach, every deep learning model in this section was independently trained and tested five times under identical hardware conditions. The final performance was reported as the mean value together with the standard deviation calculated from five replicated runs.

Table 6 summarizes the comparison of RMSE, MAE, MAPE and the batch-averaged physical residual ${\bar{\varepsilon }}_{\mathrm{phy}}$ for different models at three forecast horizons *t*, *t* + 3 and *t* + 15 under the optimal physical-constraint weight configuration.

As listed in Table 6, PI-Informer achieves the lowest values for all four metrics across the three forecast horizons. At the current time step *t*, compared with the best-performing baseline model, PI-Informer yields a relative RMSE reduction of 28.3%, a relative MAE reduction of 19.6%, a relative MAPE reduction of 24.8%, and a relative reduction of 21.21% in batch-averaged physical residual ${\bar{\varepsilon }}_{\mathrm{phy}}$. These results demonstrate its prominent advantages in both prediction accuracy and physical consistency. As the prediction step increases to *t* + 3, the difficulty of multi-step prediction rises and the errors of all models increase evidently. Nevertheless, PI-Informer exhibits a considerably smaller error increment than baseline models, with an RMSE of 0.2055 ± 0.0042 mm, representing a 32.5% reduction compared with the optimal baseline. At the long-range horizon *t* + 15, the errors of all models decrease moderately relative to *t* + 3. The RMSE of PI-Informer remains at 0.1834 ± 0.0039 mm, which is still 30.3% lower than that of the best baseline. The above results reveal that PI-Informer can extract local transient features and long-term temporal dependencies simultaneously from 2 s-long historical sequences and effectively capture the evolutionary law of molten-pool heat accumulation. Notably, the prediction error of PI-Informer at *t* + 15 is even lower than the errors of all baseline models at time *t*. This phenomenon indicates that the 1D-CNN local feature extraction module together with the physically constrained loss can significantly suppress error accumulation during long-horizon prediction. Furthermore, most metrics of PI-Informer present smaller standard deviations than those of baseline competitors, which further confirms favourable convergence stability and robustness against random parameter initialization.

**Figure 13:**
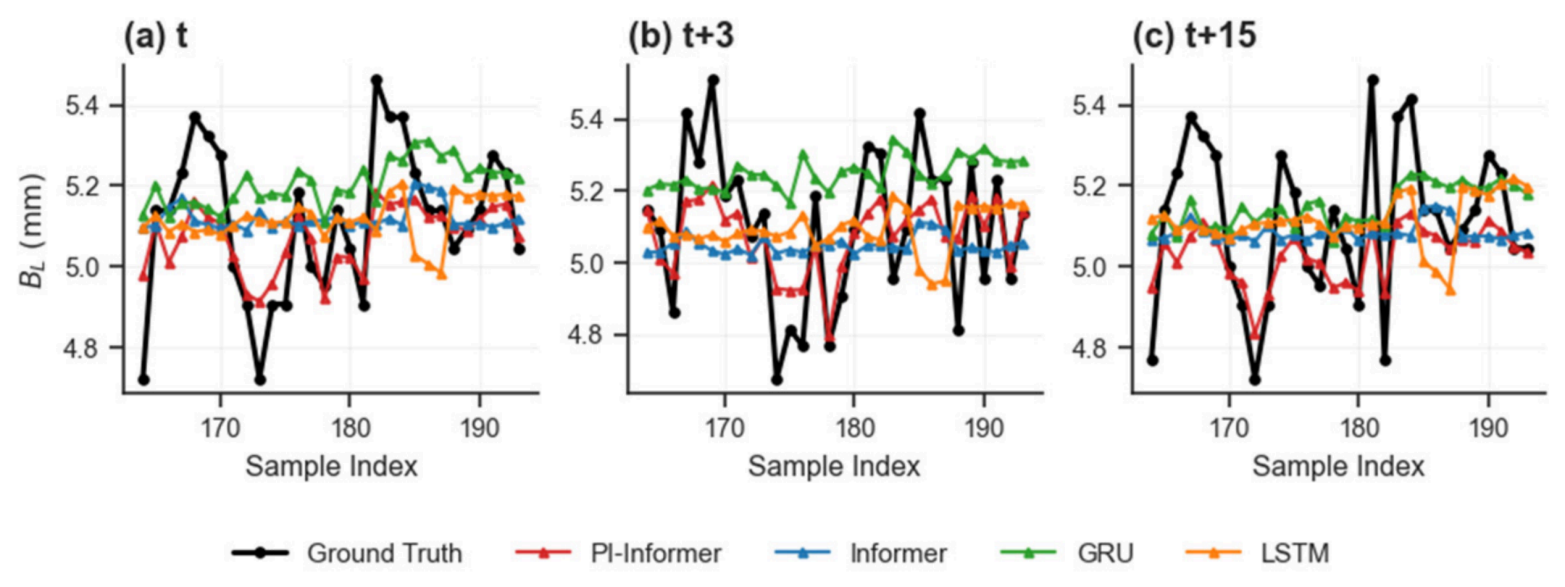
Prediction curves of comparative experiments for typical weld beads. (**a**) Prediction horizon t; (**b**) prediction horizon \(t + 3\); (**c**) prediction horizon \(t + 15\).

Figure 13 illustrates the comparison of side-wall root weld-width prediction curves of the four models at *t*, *t* + 3 and *t* + 15 for a representative weld bead under optimal physical constraints, where the black solid line denotes ground-truth values. It can be observed that the prediction curves of PI-Informer show the highest overall consistency with ground-truth values. Especially for mutation regions, wave peaks and wave troughs, PI-Informer can respond rapidly and precisely follow real changing trends. By contrast, standard Informer, GRU and LSTM generally suffer from obvious prediction lag at trend-turning points. Such model deviations are further aggravated as the prediction horizon grows. At *t* + 15, remarkable phase shift and amplitude attenuation are observed in baseline outputs, which tend to be over-smoothed and fail to reconstruct real oscillation characteristics.

**Figure 14:**
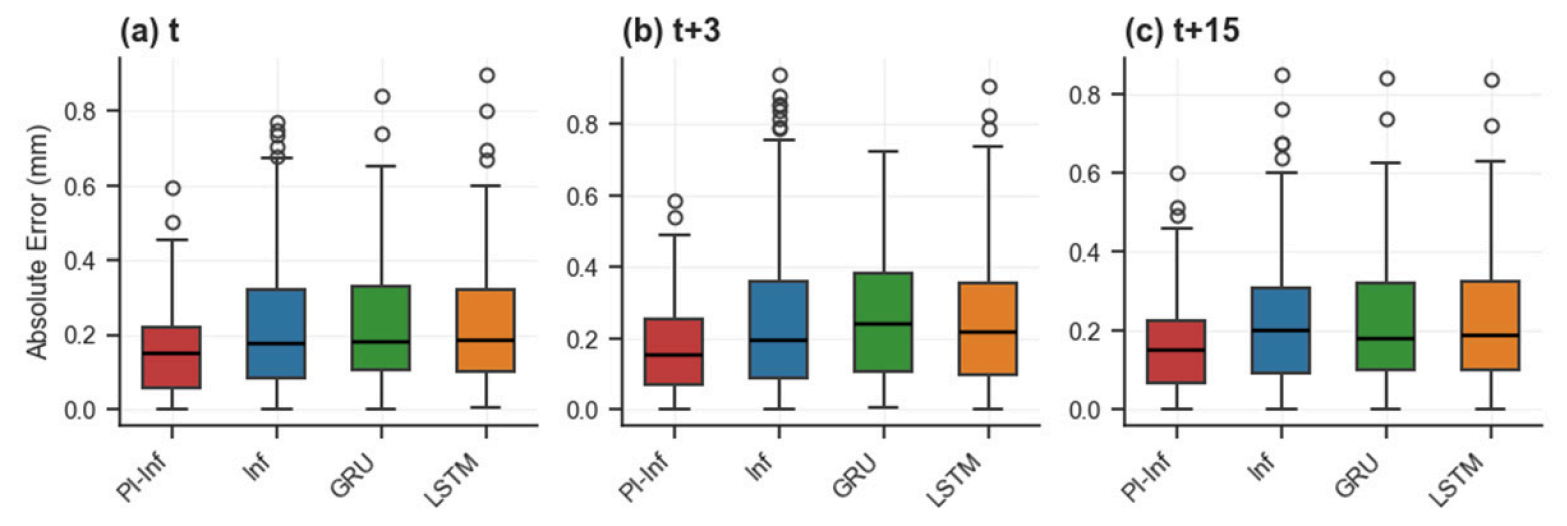
Boxplots of absolute errors for each model in comparative experiments. (**a**) Prediction horizon t; (**b**) prediction horizon \(t + 3\); (**c**) prediction horizon \(t + 15\). Comparison among PI-Inf (PI-Informer), Inf (Informer), GRU and LSTM.

Figure 14 presents box-and-whisker plots of absolute prediction errors on the test set for all competing models at the three forecast horizons. Across *t*, *t* + 3 and *t* + 15, PI-Inf (PI-Informer) consistently obtains the lowest median error with narrower interquartile boxes and more concentrated error distributions. This demonstrates that the proposed model delivers lower overall prediction error together with excellent stability and robustness. In addition, LSTM, GRU and standard Informer generate numerous high-error outlier samples and are prone to large prediction deviations. Both the quantity and magnitude of outliers produced by PI-Informer are substantially reduced compared with baseline models. It proves that the introduction of physically constrained loss improves average prediction accuracy and imposes effective constraints on model outputs. Predictions are confined within reasonable physical ranges, and extreme abnormal predictions are suppressed accordingly.

**Figure 15:**
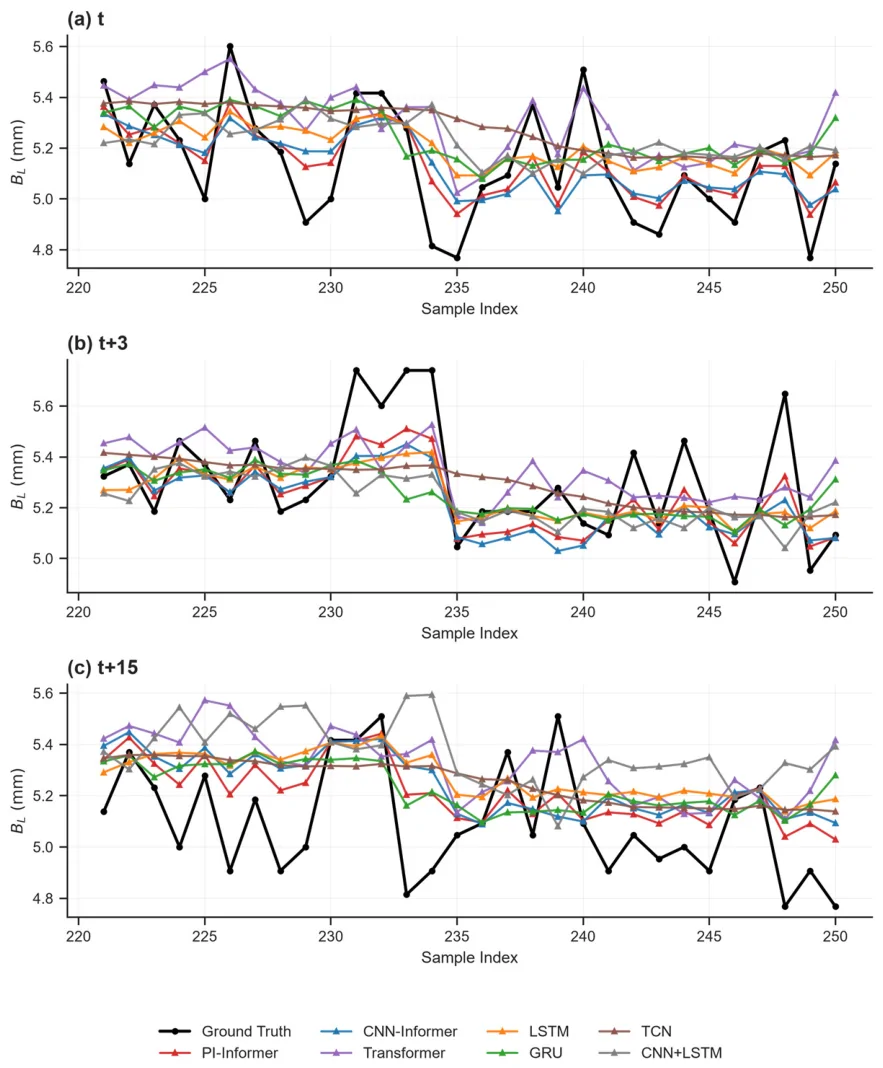
Extended comparison experiments. (**a**) Prediction horizon t; (**b**) prediction horizon \(t + 3\); (**c**) prediction horizon \(t + 15\).

To enrich our baseline comparison suite, we have added three additional competitive multivariate time-series forecasting models for comparative experiments: vanilla Transformer, Temporal Convolutional Network (TCN), and CNN-LSTM hybrid architecture. The extended comparison is performed on newly collected experimental samples independent of the original dataset. All models are trained and evaluated under identical experimental settings with five independent repeated runs. Figure 15 presents the extended comparison of predicted side-wall root fusion widths versus ground truth across different forecast horizons *t*, *t* + 3, *t* + 15 for various deep learning models. The red line with circular markers denotes PI-Informer, and the remaining lines correspond to the selected baseline models. The results indicate that PI-Informer achieves closer tracking of transient fluctuations and lower prediction errors across all horizons compared with the baseline models.

Based on the above analysis, the proposed PI-Informer outperforms mainstream time-series models in terms of prediction accuracy and physical consistency, which verifies its effectiveness and superiority for short-term trend prediction of side-wall root weld width.

## 6. Conclusions

In the prediction of side-wall root weld width for ultra-narrow gap arc welding, conventional pure data-driven models generally suffer from the absence of welding physical-mechanism constraints. These models can only realize single-step prediction at the current time step, which fails to satisfy the look-ahead regulation requirements of intelligent welding control systems. Accordingly, a PI-Informer multi-step prediction model embedded with welding thermal-balance mechanism constraints is proposed in this paper. Combining data-driven learning with welding domain prior knowledge, the model achieves multi-step prediction of weld width with high prediction accuracy and physical rationality via multi-scale local feature extraction and physical loss regularization. Systematic ablation experimental results reveal that there exists an optimal trade-off interval for the physical-constraint weight. Moderate introduction of thermal-balance physical constraints can effectively correct model fitting bias and considerably reduce the physical residual of time-series prediction. By contrast, excessively strengthened physical constraints will suppress the feature-learning capability of data-driven approaches. The optimal balance between prediction accuracy and physical consistency is achieved under the optimal weight condition of *β* = 0.05. Furthermore, the multi-scale 1D-CNN module can effectively capture transient characteristics such as metal transfer and arc fluctuation within welding electrical signals. It compensates for the deficiency that global attention in the vanilla Informer is insensitive to local abrupt changes, and substantially improves error stability and robustness across all prediction horizons. Comparative experiments verify that the proposed model exhibits obvious advantages over LSTM, GRU and vanilla Informer in quantitative metrics including MAE, RMSE and MAPE. Meanwhile, optimal physical consistency is maintained for prediction outputs, which enables better agreement with the thermal-evolution physical laws of ultra-narrow gap welding. The proposed PI-Informer overcomes the drawbacks of traditional pure data-driven models, such as poor physical interpretability, severe error accumulation in long-horizon prediction and lagging dynamic response. It can accurately track transient fluctuations during welding processes and provide a reliable modelling scheme for dynamic quality monitoring, process evaluation and look-ahead intelligent regulation of ultra-narrow gap arc welding.

Nevertheless, despite the favourable prediction performance achieved under laboratory conditions, the proposed method still has certain limitations. Model training and validation are performed under fixed equipment and stable welding conditions, where strong disturbances, variable-parameter welding scenarios, and unsteady molten-pool behaviours are not fully considered. In addition, the physical-constraint weight adopts an offline optimal value and lacks adaptive adjustment capability. For future work, adaptive physical constraint strategies will be further developed, and multi-working-condition datasets will be utilized to improve the generalization ability of the model. Furthermore, the established multi-step prediction model can be embedded into a closed-loop control system to realize adaptive intelligent regulation for ultra-narrow gap welding quality.

The proposed model is validated under laboratory conditions using Q235B steel. Its generalization ability across different base-material grades needs further experimental verification, which will be investigated in our future pipeline-steel welding research. It should be noted that dataset partitioning in this paper is performed on a weld-pass basis. Training, validation and test sets are divided by independent weld beads instead of random sample-point shuffling. The test set consists of entirely unseen weld passes during model training, which can partially reflect the model’s generalization for different welding runs under the same material system. Future work will expand datasets covering multiple material grades and broader process windows to further verify cross-sample generalization performance.

## Figures and Tables

**Table 1:** Ideal working regions under different groove widths.

*G_w_* (mm)	*V* (mm/s)	*V_f_* (mm/s)	*U* (V)	*I* (A)	*κ*_ref_	*χ*_ref_
4.9	7.0~8.0	110~115	21.5~22.0	215~245	0.88	0.68
5.0	7.5~8.5	115~118	22.0~22.5	230~258	0.85	0.66
5.2	8.0~8.3	115~120	22.0~22.5	240~265	0.84	0.65
5.3	8.0~8.5	115~122	22.0~22.5	240~265	0.85	0.67
5.5	8.5~9.0	115~125	22.5~22.5	255~280	0.86	0.68
6.2	9.0~10.0	120~125	22.5~23.5	270~300	0.85	0.67

**Table 2:** Experimental parameters of the UNG-AW.

Exp. No	*G_w_* (mm)	*U* (V)	*V_f_* (mm/s)	*V* (mm/s)
Exp. No. 1	4.9	21.0	110	7.0
Exp. No. 2	5.0	22.0	115	8.0
Exp. No. 3	5.2	22.0	110	7.0
Exp. No. 4	5.3	22.0	115	8.0
Exp. No. 5	5.5	22.5	120	8.5
Exp. No. 6	6.2	24.5	125	8.5

**Table 3:** PSO-optimized hyperparameter configurations for comparative models.

Model	Hyperparameter	Search Range	Optimized Value
PI-Informer	Learning rate	[1 × 10^−5^, 1 × 10^−3^]	0.0005
	Batch size	{16, 32, 64, 128}	64
	Embedding dimension	{128, 256, 512}	256
Vanilla Informer	Learning rate	[1 × 10^−5^, 1 × 10^−3^]	0.0004
	Batch size	{16, 32, 64, 128}	64
	Embedding dimension	{128, 256, 512}	256
LSTM	Learning rate	[1 × 10^−5^, 1 × 10^−3^]	0.0007
	Batch size	{16, 32, 64, 128}	32
	Number of hidden units	{64, 128, 256}	128
GRU	Learning rate	[1 × 10^−5^, 1 × 10^−3^]	0.0006
	Batch size	{16, 32, 64, 128}	32
	Number of hidden units	{64, 128, 256}	128
Transformer	Learning rate	[1 × 10^−5^, 1 × 10^−3^]	0.00045
	Batch size	{16, 32, 64, 128}	64
	Embedding dimension	{128, 256, 512}	256
TCN	Learning rate	[1 × 10^−5^, 1 × 10^−3^]	0.0008
	Batch size	{16, 32, 64, 128}	32
	Filter number	{64, 128, 256}	128
CNN-LSTM	Learning rate	[1 × 10^−5^, 1 × 10^−3^]	0.00065
	Batch size	{16, 32, 64, 128}	64
	LSTM hidden units	{64, 128, 256}	128

**Table 4:** Comparison of prediction performance in β ablation experiments.

*β*	Horizon	RMSE (mm)	MAE (mm)	MAPE (%)	${\bar{\varepsilon }}_{\mathrm{phy}}$ (mm)
0.00	*t*	0.2287 ± 0.0033	0.1896 ± 0.0027	3.5521 ± 0.0515	0.3130 ± 0.0049
	*t* + 3	0.2572 ± 0.0034	0.2130 ± 0.0027	3.9910 ± 0.0520	0.3254 ± 0.0051
	*t* + 15	0.2209 ± 0.0041	0.1820 ± 0.0030	3.4230 ± 0.0641	0.3039 ± 0.0071
0.01	*t*	0.2239 ± 0.0024	0.1844 ± 0.0027	3.4712 ± 0.0370	0.2794 ± 0.0039
	*t* + 3	0.2409 ± 0.0026	0.1989 ± 0.0019	3.7320 ± 0.0409	0.2790 ± 0.0057
	*t* + 15	0.2251 ± 0.0041	0.1860 ± 0.0018	3.4913 ± 0.0641	0.3031 ± 0.0083
0.02	*t*	0.2341 ± 0.0029	0.1932 ± 0.0031	3.6330 ± 0.0460	0.2980 ± 0.0036
	*t* + 3	0.2552 ± 0.0026	0.2106 ± 0.0015	3.9607 ± 0.0349	0.2820 ± 0.0062
	*t* + 15	0.2359 ± 0.0023	0.1949 ± 0.0029	3.6616 ± 0.0460	0.2673 ± 0.0064
0.04	*t*	0.2347 ± 0.0043	0.2117 ± 0.0035	3.6406 ± 0.0672	0.2742 ± 0.0058
	*t* + 3	0.2384 ± 0.0058	0.1969 ± 0.0046	3.7003 ± 0.0864	0.2719 ± 0.0070
	*t* + 15	0.2560 ± 0.0035	0.2116 ± 0.0024	3.9704 ± 0.0535	0.2801 ± 0.0048
0.045	*t*	0.1990 ± 0.0028	0.1750 ± 0.0025	3.0825 ± 0.0431	0.2506 ± 0.0064
	*t* + 3	0.2155 ± 0.0033	0.1969 ± 0.0036	3.3308 ± 0.0518	0.2559 ± 0.0100
	*t* + 15	0.1958 ± 0.0023	0.1858 ± 0.0030	2.9816 ± 0.0352	0.2452 ± 0.0123
0.05	*t*	0.1879 ± 0.0026	0.1526 ± 0.0021	2.8427 ± 0.0396	0.1839 ± 0.0047
	*t* + 3	0.2071 ± 0.0031	0.1701 ± 0.0025	3.1546 ± 0.0479	0.2046 ± 0.0092
	*t* + 15	0.1828 ± 0.0021	0.1500 ± 0.0016	2.8083 ± 0.0311	0.1921 ± 0.0041
0.055	*t*	0.2085 ± 0.0029	0.1706 ± 0.0024	3.2330 ± 0.0459	0.2674 ± 0.0048
	*t* + 3	0.2336 ± 0.0038	0.1928 ± 0.0032	3.6205 ± 0.0594	0.2590 ± 0.0080
	*t* + 15	0.2142 ± 0.0062	0.1768 ± 0.0051	3.3212 ± 0.0974	0.2545 ± 0.0064
0.10	*t*	0.2378 ± 0.0063	0.1932 ± 0.0051	3.6314 ± 0.0963	0.2768 ± 0.0062
	*t* + 3	0.2521 ± 0.0016	0.2047 ± 0.0013	3.8421 ± 0.0244	0.3165 ± 0.0097
	*t* + 15	0.2308 ± 0.0042	0.1883 ± 0.0034	3.5430 ± 0654	0.3017 ± 0.0082

**Table 5:** 1D-CNN ablation experiment error metrics.

Model	Horizon	RMSE (mm)	MAE (mm)	MAPE (%)	${\bar{\varepsilon }}_{\mathrm{phy}}$ (mm)
PI-Informer (w/CNN)	*t*	0.1879 ± 0.0025	0.1526 ± 0.0021	2.8429 ± 0.0396	0.1841 ± 0.0047
	*t* + 3	0.2072 ± 0.0034	0.1709 ± 0.0026	3.1562 ± 0.0479	0.2044 ± 0.0092
	*t* + 15	0.1880 ± 0.0020	0.1528 ± 0.0017	2.8677 ± 0.0311	0.1954 ± 0.0041
PI-Informer (w/o CNN)	*t*	0.2619 ± 0.0031	0.2120 ± 0.0035	4.0695 ± 0.0425	0.2576 ± 0.0052
	*t* + 3	0.2538 ± 0.0047	0.2066 ± 0.0032	3.9250 ± 0.0526	0.2623 ± 0.0098
	*t* + 15	0.2102 ± 0.0036	0.1986 ± 0.0029	3.2120 ± 0.0341	0.2549 ± 0.0049

**Table 6:** Analysis of prediction performance for comparative experiments.

Horizon	Metric	PI-Informer	Informer	LSTM	GRU
*t*	RMSE (mm)	0.1896 ± 0.0031	0.2621 ± 0.0054	0.2704 ±0.0058	0.2740 ±0.0062
	MAE (mm)	0.1577 ± 0.0029	0.2164 ± 0.0042	0.2216 ± 0.0053	0.2209 ± 0.0059
	MAPE( %)	2.8381 ± 0.0228	4.0311 ± 0.0314	4.1381 ± 0.0377	4.1035 ± 0.0343
	${\bar{\varepsilon }}_{\mathrm{phy}}$ (mm)	0.1842 ± 0.0229	0.2338 ± 0.0312	0.2381 ± 0.0298	0.2413 ± 0.0315
*t* + 3	RMSE (mm)	0.2055 ± 0.0042	0.3051 ± 0.0061	0.3043 ± 0.0055	0.3056 ± 0.0068
	MAE (mm)	0.1710 ± 0.0024	0.2418 ± 0.0039	0.2546 ± 0.0043	0.2472 ± 0.0039
	MAPE (%)	3.1603 ± 0.0571	4.4220 ± 0.0599	4.8036 ± 0.0613	4.5407 ± 0.0665
	${\bar{\varepsilon }}_{\mathrm{phy}}$ (mm)	0.2087 ± 0.0329	0.3038 ± 0.0479	0.3169 ± 0.0423	0.3191 ± 0.0437
*t* + 15	RMSE (mm)	0.1834 ± 0.0039	0.2638 ± 0.0049	0.2631 ± 0.0051	0.2659 ± 0.0047
	MAE (mm)	0.1525 ± 0.0037	0.2143 ± 0.0033	0.2155 ± 0.0039	0.2174 ± 0.0039
	MAPE (%)	2.9024 ± 0.0602	3.9881 ± 0.0710	3.9802 ± 0.0733	4.0217 ± 0.0630
	${\bar{\varepsilon }}_{\mathrm{phy}}$ (mm)	0.1942 ± 0.0319	0.2831 ± 0.0483	0.2648 ± 0.0522	0.2818 ± 0.0539

## Data Availability

The raw welding experimental data are not publicly available due to confidentiality provisions of the ongoing internal laboratory project and the massive volume of high-frequency sensing signals they contain. Processed data that support the findings of this study are available from the corresponding author upon reasonable request.
